# Exposome in ischaemic heart disease: beyond traditional risk factors

**DOI:** 10.1093/eurheartj/ehae001

**Published:** 2024-01-18

**Authors:** Rocco A Montone, Massimiliano Camilli, Camilla Calvieri, Giulia Magnani, Alice Bonanni, Deepak L Bhatt, Sanjay Rajagopalan, Filippo Crea, Giampaolo Niccoli

**Affiliations:** Department of Cardiovascular Medicine, Fondazione Policlinico Universitario A. Gemelli IRCCS, L.go A. Gemelli, 1, 00168 Rome, Italy; Department of Cardiovascular Medicine, Fondazione Policlinico Universitario A. Gemelli IRCCS, L.go A. Gemelli, 1, 00168 Rome, Italy; Department of Cardiovascular and Pulmonary Sciences, Catholic University of the Sacred Heart, Rome, Italy; Sapienza University, Rome, Italy; Department of Medicine, University of Parma, Parma, Italy; Department of Cardiovascular Medicine, Fondazione Policlinico Universitario A. Gemelli IRCCS, L.go A. Gemelli, 1, 00168 Rome, Italy; Mount Sinai Heart, Icahn School of Medicine at Mount Sinai, New York, NY, USA; Cardiovascular Research Institute, Case Western Reserve University, Cleveland, OH 44106, USA; Department of Cardiovascular Medicine, Fondazione Policlinico Universitario A. Gemelli IRCCS, L.go A. Gemelli, 1, 00168 Rome, Italy; Department of Cardiovascular and Pulmonary Sciences, Catholic University of the Sacred Heart, Rome, Italy; Department of Medicine, University of Parma, Parma, Italy

**Keywords:** Ischaemic heart disease, Risk factors, Atherosclerosis, Pollution, Mental stress

## Abstract

Ischaemic heart disease represents the leading cause of morbidity and mortality, typically induced by the detrimental effects of risk factors on the cardiovascular system. Although preventive interventions tackling conventional risk factors have helped to reduce the incidence of ischaemic heart disease, it remains a major cause of death worldwide. Thus, attention is now shifting to non-traditional risk factors in the built, natural, and social environments that collectively contribute substantially to the disease burden and perpetuate residual risk. Of importance, these complex factors interact non-linearly and in unpredictable ways to often enhance the detrimental effects attributable to a single or collection of these factors. For this reason, a new paradigm called the ‘exposome’ has recently been introduced by epidemiologists in order to define the totality of exposure to these new risk factors. The purpose of this review is to outline how these emerging risk factors may interact and contribute to the occurrence of ischaemic heart disease, with a particular attention on the impact of long-term exposure to different environmental pollutants, socioeconomic and psychological factors, along with infectious diseases such as influenza and COVID-19. Moreover, potential mitigation strategies for both individuals and communities will be discussed.

## Graphical Abstract

**Graphical Abstract:**
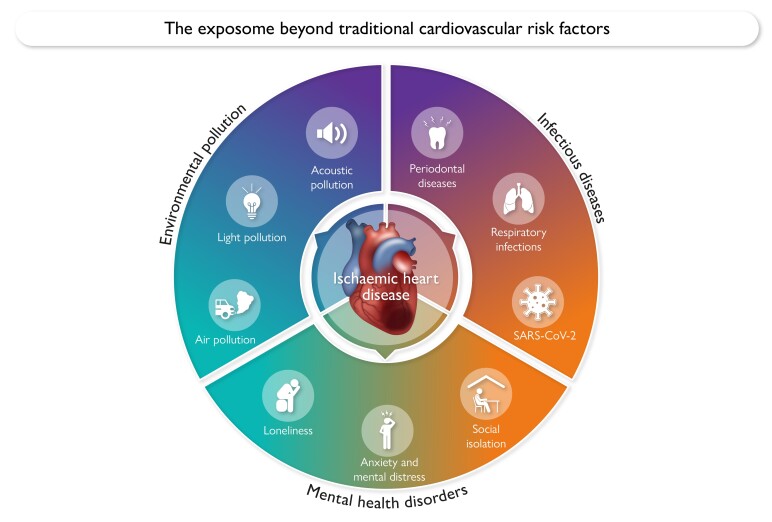
Major non-traditional risk factors for ischaemic heart disease, including environmental pollution, mental health disorders, and infectious diseases. SARS-CoV-2, severe acute respiratory syndrome coronavirus 2.

**This paper was guest edited by Prof. Thomas Lüscher**

## Introduction

Ischaemic heart disease (IHD) is a major cause of morbidity and mortality worldwide, classically triggered by the deleterious effects of risk factors on endothelial cells.^1–3^ Preventive measures based on traditional risk factors identified in the Framingham Heart Study^4,5^ such as arterial hypertension, diabetes, dyslipidaemia, and smoking have decreased IHD incidence but the latter remains the number one killer worldwide; thus, other contributors must be addressed in order to further reduce the disease burden.^6^ Moreover, among 62 048 patients with first-presentation ST-elevation myocardial infarction, 15% without standard modifiable cardiovascular risk factors (defined as SMuRFs) shows significantly increased risk of all-cause mortality compared with those with at least one modifiable risk factor.^7^ This observation calls to action for the identification of yet undiscovered aetiologies in the IHD arena.

Three environmental broad domains play a major role in IHD. In particular, research into natural, built, and social environments has advanced the importance of known factors (*Table 1*),^8–30^ although still many others can contribute to the genesis of ischaemic and metabolic diseases. These factors cannot be directly modified by treating traditional cardiovascular risk factors, hence current prevention strategies focused on these latter could fail.^31^ Thus, the recent focus on non-traditional risk factors, including pollution (air, water, soil, and chemical exposures), mental stress, depression and social isolation, as well as infectious *noxae*^31,32^ is quite appropriate.

**Table 1:** Main studies prospectively investigating the impact of non-traditional cardiovascular risk factors on ischaemic heart disease

Study	Population	Exposure	Outcomes	Principal findings
Air pollution				
Yusuf S, *et al.* Lancet 2020 (PURE)^8^	155 722 participants without a prior history of CV disease from 21 HICs, MICs, or LICs	Household air pollution	Composite of CV disease events (CV death, MI, stroke, and HF) and mortality	Household air pollution was associated with a higher risk of CV disease [(HICs: HR: 1.00, 95% CI: 1.00–1.00); (MICs: HR 1.02, 95% CI: 0.92–1.13); (LICs: HR 1.23, 95% CI: 1.01–1.49)] and death [(HICs: HR 1.00, 95% CI: 1.00–1.00); (MICs: HR 1.20, 95% CI: 1.08–1.34); (LICs: HR 1.22, 95% CI: 1.04–1.43)] particularly among LICs.
Downward GS, *et al.* Environ Health Perspect. 2018^9^	33 831 Dutch residents without history of CV disease	Long-term UFP (smaller than 100 nm)	CVD and MI	Long-term UFP exposure was associated with an increased risk for all incident CVD [HR 1.18 per 10 000particles/cm^3^; 95% CI: 1.03, 1.34], and MI (HR 1.34; 95% CI: 1.00–1.79).
Turner MC, *et al.* Am J Respir Crit Care Med. 2016 (CPS II II)^10^	669 046 from 50 USA, the District of Columbia, and Puerto Rico	Chronic ambient O3	CV and all-cause mortality	Significant positive associations between O3 and all-cause (HR per 10 ppb 1.02; 95% CI: 1.01–1.04), as well as cardiovascular (HR per 10 ppb 1.03; 95% CI: 1.01–1.05) mortality.
Kaufman JD, *et al.* Lancet. 2016 (MESA Air)^11^	6795 participants from 6 metropolitan areas in the USA	Long-term exposure to PM2.5 and NOX	Coronary calcium score by computed tomography	For each 5 μg/m^3^ increase in PM2.5, coronary calcium progressed by 4.1 Agatston units per year (95% CI: 1.40–6.80) and for each 40 ppb NOX coronary calcium progressed by 4.8 Agatston units per year (95% CI: 0.90–8.70).
Cesaroni G, *et al.* BMJ. 2014 (ESCAPE)^12^	11 European cohorts, including 100 166 people free from previous coronary events	Long-term (average of 11.5 years) exposure to particulate matter PM2.5 and PM10	Meta-analysis of the cohort specific results for coronary events	A 5 μg/m^3^ increase in estimated annual mean PM2.5 was associated with a 13% increased risk of coronary events (HR 1.13, 95% CI 0.98–1.30), and a 10 μg/m^3^ increase in estimated annual mean PM10 was associated with a 12% increased risk of coronary events (HR 1.12, 95% CI 1.01–1.25) with no evidence of heterogeneity between cohorts.
Pope CA 3rd, *et al.* JAMA 2002 (CPS-II)^13^	319 000 adults from 51 US metropolitan areas	Fine particulate air pollution	Cardiopulmonary mortality and overall mortality	Elevation in fine particulate air pollution was associated with cardiopulmonary mortality (adj. RR for a 10 μg/m^3^ change in PM2.5 1.09, 95% CI: 1.03–1.16) and all cause of death (adj. RR 1.06, 95% CI: 1.02–1.11).
Light pollution				
Xu Yx *et al.* Environ pollut. 2022^14^	484 Chinese young adults	Light at night (LAN)	Cardiometabolic (CM) risk, fasting insulin, total cholesterol, triglyceride, and LDL cholesterol	Exposure to higher bedroom LAN intensity is associated with 1.47 unit increase in CM-risk score (95% CI: 0.69–2.25; *P* < .001). Besides, post-bedtime light exposure was associated with elevated fasting insulin (PBL-1h: β = 0.06, 95% CI: 0.01–0.10; PBL-4h: β = 0.33, 95% CI: 0.19–0.47) and HOMA-IR (PBL-1h: β = 0.013, 95% CI: 0–0.03; PBL-4h: β = 0.07, 95% CI: 0.04–0.11) while pre-awake light exposure was associated with elevated total cholesterol (PAL-1h: β = 0.03, 95% CI: 0.02–0.04; PAL-2h: β = 0.02, 95% CI: 0.01–0.03), triglyceride (PAL-1h: β = 0.015, 95% CI: 0.01–0.02; PAL-2h: β = 0.01, 95% CI: 0–0.02) and low-density lipoprotein cholesterol (PAL-1h: β = 0.02, 95% CI: 0.01–0.03; PAL-2h: β = 0.02, 95% CI: 0.01–0.03).
Sun S. *et al.* Eur Heart J. 2021^15^	58 692 Chinese elders, resident in the 18 districts of Hong Kong	Outdoor light at night (followed for a median of 11 years)	Risk of CHD hospitalization and mortality	An interquartile range (60.0 nW/cm^2^/sr) increase in outdoor light at night was associated with an HR of 1.11 (95% CI: 1.03, 1.18) for CHD hospitalizations and 1.10 (95% CI: 1.00, 1.22) for CHD mortality.
Obayashi K. *et al.* Environ Int. 2019^16^	989 community-dwelling elderly people	Bedroom light intensity during the night-time	Carotid artery intima-media thickness (IMT)	The highest LAN group exhibited a significant increase in mean carotid IMT (adjusted β, 0.028; 95% CI, 0.005–0.052; *P* = .019) compared with the lowest LAN quartile group.
Obayashi K. *et al.* Chronobiol Int. 2014^17^	528 home-dwelling Japanese elderly	Light at night (LAN)	Blood pressure (BP)	Increase in systolic and diastolic blood pressure by 3.7% and 4.5% (4.3 and 3.0 mmHg), respectively, for a 5 lux [1 lux = 1 lumen/m^2^; 1 lumen is equivalent to ∼0.1 W (bulb) or 0.01 W (LED)] increase in outdoor light exposure at night.
Acoustic pollution				
Saucy A. *et al.* Eur Heart J. 2021^18^	24 886 cases of death from cardiovascular disease (CVD) from the Swiss National Cohort	Night-time aircraft noise	Case-crossover for all causes of CVD	For night-time deaths, exposure levels 2 h preceding death were significantly associated with mortality for all causes of CVD [OR = 1.44 (1.03–2.04) for the highest exposure group (average A-weighted equivalent continuous sound pressure level (LAeq) LAeq > 50 dB vs. < 20 dB)].
Kupcikova Z. *et al.* Eur Heart J. 2021^19^	502 651 individuals from the UK Biobank	Road traffic noise	Cardiovascular disease risk factors: systolic (SBP) and diastolic blood pressure (DBP), C-reactive protein, triglycerides, glycated haemoglobin, and self-reported hypertension	Exposure to road traffic (Lden) > 65 dB[A], as compared to ≤55 dB[A], was associated with 0.77% [95% confidence interval (CI) 0.60%, 0.95%], 0.49% (95% CI 0.32%, 0.65%), 0.79% (95% CI 0.11%, 1.47%), and 0.12% (95% CI −0.04%, 0.28%) higher SBP, DBP, triglycerides, and glycated haemoglobin, respectively.
Osborne MT. *et al.* Eur Heart J. 2020^20^	498 adults without CVD from Boston, MA, USA	Transportation noise	Major adverse cardiovascular disease events (MACE)	Increase of 5 dBA predicted MACE [hazard ratio (95% confidence interval, CI) 1.341 (1.147–1.567), *P* < .001.
Correia AW. *et al.* BMJ 2013^21^	6 027 363 elderly people, residing in the 2218 zip codes close to the 89 airports	Aircraft noise	Five cause specific cardiovascular hospital admissions: heart failure, heart rhythm disturbances, cerebrovascular events, ischaemic heart disease, and peripheral vascular disease	An increase of 10 dB was associated with an increase of 2.9% (95% confidence interval 0.8% to 5.0%) in hospital admission rate.
Social stress				
Gan Y, Gong Y, *et al.* BMC Psychiatry. 2014^22^	893 850 participants from 30 prospective cohort studies conducted in North America, Western Europe, and Asia	Depression	Meta-analysis of the specific results for CHD and MI	Depression was associated with an increased risk of MI (RR 1.30; 95% CI: 1.22–1.40) and incident CHD (RR 1.30; 95% CI: 1.18–1.44). These associations remained significant after adjustment for sociodemographic factors and health behaviours.
Nabi H, *et al.* Eur Heart J. 2013^23^	7268 men and women from the British Whitehall II cohort study	Stress	Coronary death, non-fatal MI	After adjustment for sociodemographic characteristics, participants who reported at baseline that stress has affected their health ‘a lot or extremely’ had 2.12 times higher (95% CI: 1.52–2.98) risk of coronary death or incident non-fatal MI when compared with those who reported no effect of stress on their health.
Richardson S, *et al.* Am J Cardiol. 2012^24^	118 696 participants from 6 prospective observational cohort studies	Perceived stress regardless of cause	Meta-analysis of the specific results for CHD	High vs. low perceived stress for incident CHD is associated with a risk ratio of 1.27 (95% CI 1.12–1.45) for incident CHD.
Russ TC, *et al.* BMJ. 2012^25^	Meta-analysis of 10 prospective cohort studies from the Health Survey for England. 68 222 people from general population living in private households in England.	Psychological distress	CV mortality	Psychological distress was associated with increased risk of CV mortality (adj. HR for General Health Questionnaire scores of 1–3 vs. score 0: 1.29, 95% CI: 1.17–1.43; scores 4–6: 1.44, 95% CI: 1.27–1.62; and scores 7–12: 2.05, 95% CI: 1.57–2.70; *P* < .001 for trend).
Janszky I, *et al.* J Am Coll Cardiol. 2010^26^	49 321 young Swedish men	Chronic anxiety	CHD, MI	Anxiety independently predicted subsequent CHD events (HR 1.04; 95% CI: 0.70–1.54), and acute MI (HR 1.03; 95% CI: 0.65–1.65).
Infectious diseases				
Pieralli F, *et al.* BMC Infect Dis. 2021 FADOI-ICECAP Study^27^	1266 patients enrolled during hospitalization for CAP in Internal Medicine Units	CAP	In-hospital and 30-day mortality	In-hospital (12.2% vs. 4.7%, *P* < .0001) and 30-day (16.3% vs. 8.9%, *P* = .0001) mortality was higher in patients with CV complications.
Violi F, *et al.* Clin Infect Dis. 2017 SIXTUS Study Group^28^	1182 patients hospitalized for CAP	CAP	Death at 30 days	30-Day mortality was higher in patients who developed intrahospital CV events (17.6% vs. 4.5%, *P* < .001). Intrahospital CV events (HR 5.49, 95% CI, 2.91–10.38, *P* < .001) independently predicted 30-day mortality.
Cangemi R, *et al.* Am J Cardiol. 2015 SIXTUS Study Group^29^	301 consecutive patients with CAP	CAP	Death for any cause	Death was higher in patients who experienced a CV complication (32 vs. 13%, *P* < .001). Intrahospital CV, age and PSI, independently predicted overall mortality.
Corrales-Medina VF, *et al.* Circulation 2012^30^	1343 inpatients and 944 outpatients with CAP	CAP	Death at 30 days	CV complications were associated with increased risk (OR, 1.6; 95% CI, 1.04–2.5) of death at 30 days after adjustment for baseline PSI score.

CPS-II, Cancer Prevention Study II; CV, cardiovascular; HICs, high-income countries; MICs, middle-income countries; LICs, low-income countries; CV, cardiovascular; HF, heart failure; HR, hazard ratio; CI, confidence interval; RR, relative risk; PM2.5, particles measuring <2.5 μm in diameter; PM10, particles measuring <10 μm in diameter; PURE, Prospective Urban Rural Epidemiology; MI, myocardial infarction; O3, tropospheric ozone; UFP, ultrafine particles; ESCAPE, European Study of Cohorts for Air Pollution Effects; ppb, parts per billion; MESA Air, Multi-Ethnic Study of Atherosclerosis and Air Pollution; NOX, nitrogen oxides; CHD, coronary heart disease; CAP, community-acquired pneumonia; PSI, Pneumonia Severity Index score; HF, heart failure; CVD, cardiovascular disease; HOMA-IR, homeostasis model assessment for insulin resistance; PBL-1h, 1 h average light intensity after bedtime; PBL-4h, 4 h average light intensity after bedtime; PAL-1h, 1 h average light intensity before rising time; PAL-2h, 2 h average light intensity before rising time.

**Figure 1:**
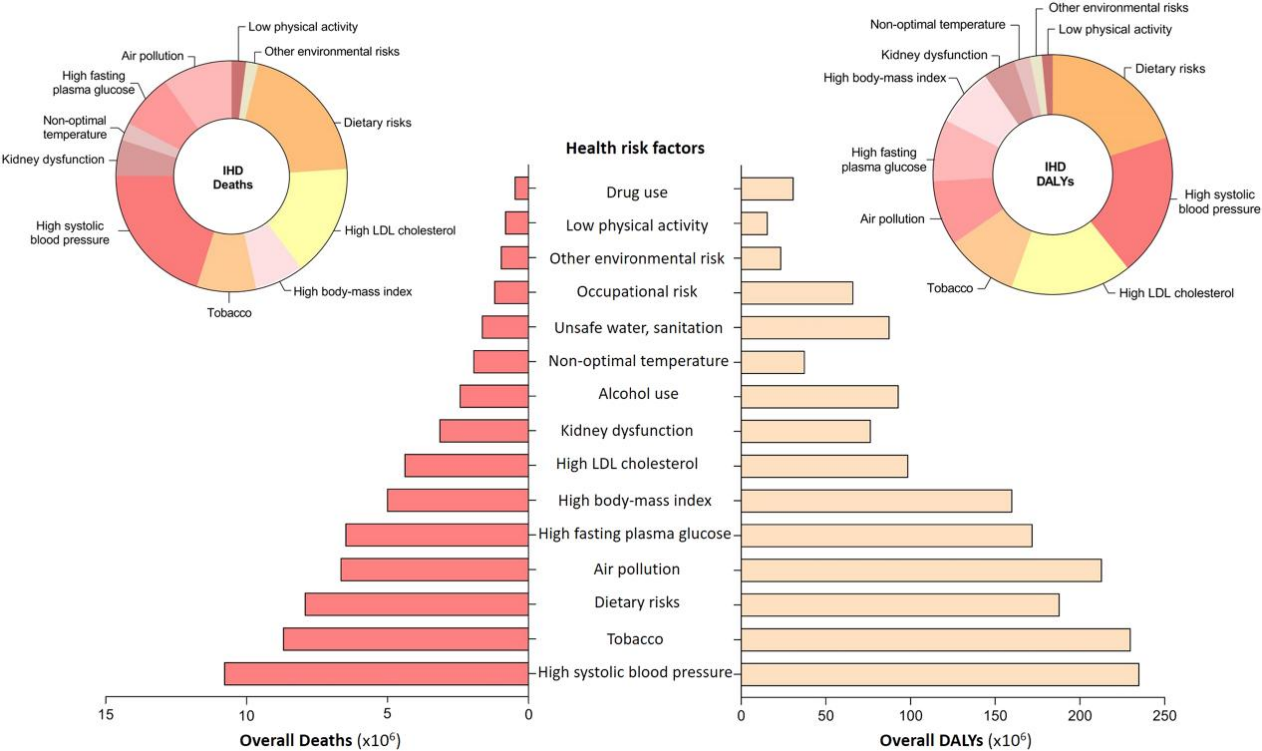
Impact of traditional and non-traditional risk factors on overall death, and death from ischaemic heart disease and DALYs. Data from the GBD 2019 reports^33^

**Figure 2:**
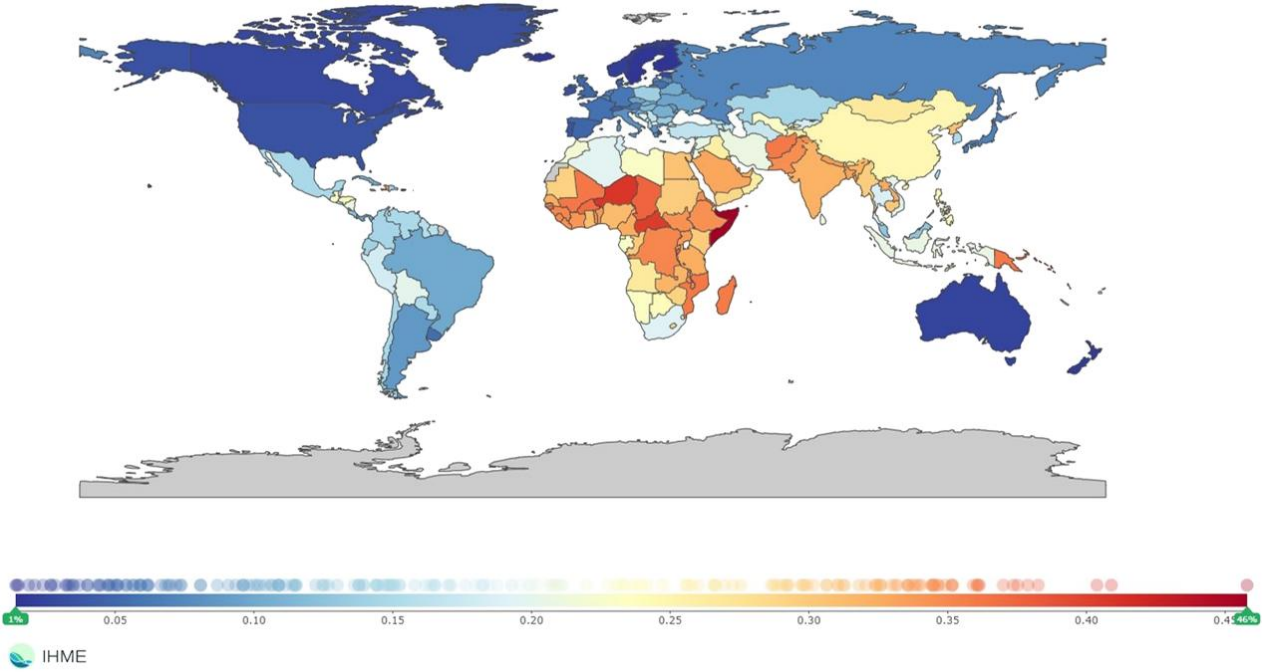
Percentage of ischaemic heart disease mortality burden attributable to air pollution for both sexes, all ages in 2019. Data from the GBD 2019 reports (Institute of Health and Metrics and Evaluation, IHME)^35^

The Global Burden of Disease (GBD) report clearly highlights the relevance of environmental stressors in determining the burden of mortality and disability-adjusted life years (DALYs), and among them, ambient air pollution has become the main environmental cause of disease and premature death worldwide, even when compared with other traditional cardiovascular risk factors (*Figure 1*).^33^ Accordingly, air pollution has been shown to reduce the global average life expectancy by 2.9 years, a reduction that is more extensive when compared with traditional cardiovascular risk factors such as tobacco smoking (2.2 years).^34^ At the same time, the effects of environmental pollution in terms of mortality are expected to increase with advancing age, due to direct aging effects, multiple comorbidities developed, including coexisting cardiovascular risk factors, and a longer exposition to environmental insults, and this burden will be particularly higher in developing countries (*Figure 2*).^35^

Environmental risk factors exhibit system interactions often with large effects attributable to non-linear interactions among them and consequent effect amplification. Thus, in the last decade, a new paradigm called the ‘exposome’ has been introduced by epidemiologists, in order to define the totality of exposure to these new risk factors in the natural, built and social environments.^36^ The exposome appears as a highly variable and dynamic entity, evolving throughout the individual lifetime. Furthermore, the exposome may represent another key player involved in determining the residual inflammatory risk,^37^ which today represents one of the main concerns to be addressed in the IHD arena, since the thrombotic and lipid risk have mostly been tackled through pharmacological treatment.

Therefore, the aim of this review is to describe the impact of non-traditional and emerging risk factors on IHD. We will focus on the role of chronic exposure to various environmental pollutants, socioeconomic and psychological determinants, and infectious diseases, including COVID-19. Furthermore, we will propose possible strategies for risk mitigation, both at individual and community levels.

## Environmental pollution

### Air pollution

Air pollution is a heterogeneous mixture of gases and particles, derived from both human and natural activities.^38,39^ Ambient particles include coarse particles with aerodynamic diameters ranging from 2.5 to 10 µm (PM10), fine particles (<2.5 µm; PM2.5), and ultrafine particles (<0.1 µm).^38,39^ The chemical composition of particles differs considerably, depending on geographical, meteorological, and source-specific variables.^40^ Usually, ambient particles include inorganic components (sulfates, nitrates, ammonium, chloride, and trace metals), elemental and organic carbon, crystal materials, biological components, and adsorbed volatile and semi-volatile organic compounds.^40^ Additionally, ambient particles can generate ambient aerosols when combined with atmospheric gases such as ozone, sulfur and nitric oxides (NO), and carbon monoxide (CO).^40^ Although a growing body of studies supports the toxicity of ultrafine and coarse particles, most available evidence recognizes PM2.5 as the primary air pollutant causing deleterious effects on human organism.^40,41^

The GBD study estimated that in 2019, ∼7.0 million deaths worldwide were directly attributable to air pollution, of which 4.1 million to ambient air pollution and 2.3 million to the household component.^1^ Moreover, air pollution is recognized as a leading cause of excess mortality and loss of life expectancy, particularly through cardiovascular disease.^34^

Of note, both short-term and chronic exposure to pollutants are responsible for increased relative risk of cardiovascular events, such as heart failure hospitalizations, cardiac arrest, arrhythmias, ischaemic stroke, and above all myocardial infarction (MI).^38,39^ Indeed, high levels of PM2.5 may enhance coronary atherosclerosis as well as plaque destabilization.^42–44^ Furthermore, recent evidence suggests that in patients with recurrent acute coronary syndrome (ACS), higher long-term PM2.5 exposure is associated with impaired plaque healing.^45^

In human studies, plasma oxidized low-density lipoprotein (LDL) concentration was positively associated with chronic air pollution exposition and this correlation was not influenced neither by conventional cardiovascular risk factors, nor lipid lowering drugs, suggesting an independent role of pollutants on the atherosclerotic process.^46^ In addition, recent data show that short- and medium-term exposures to higher levels of pollutants, in particular PM2.5, are associated with significant impairments in high-density lipoprotein functionality, as well as elevations in oxidized LDL, metrics of systemic inflammation C-reactive protein, and oxidative stress.^47,48^ In this setting, statins are only partly able to reverse levels of cholesterol, oxidative stress, and inflammatory response in mice.^49^

**Figure 3:**
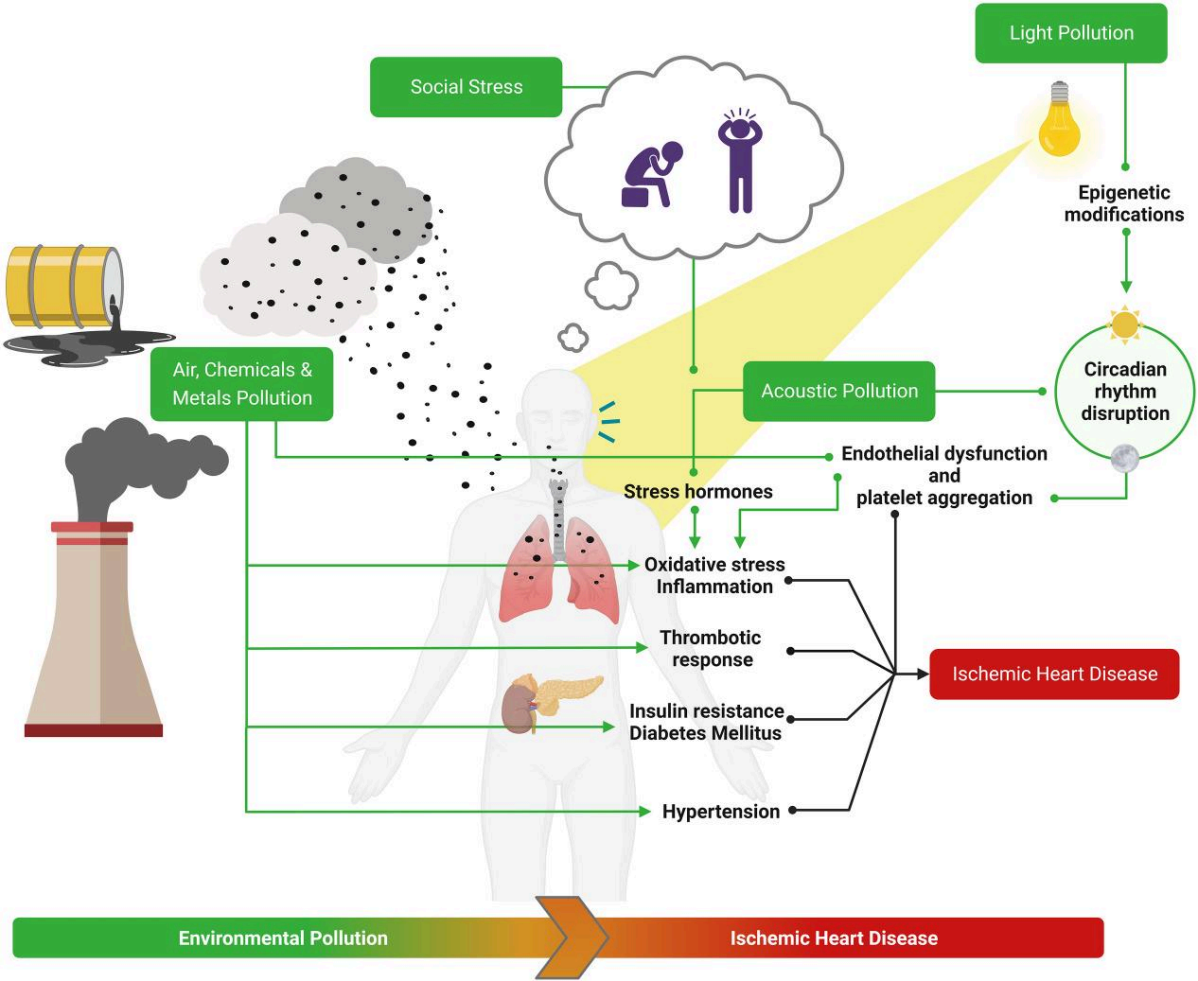
Air, light, and acoustic pollution represent relevant non-traditional risk factors for ischaemic heart disease. Air pollution through biological intermediates induces oxidative stress, inflammation which can generate insulin resistance and diabetes mellitus. Also, acoustic and light pollution and social stress enhance oxidative stress and inflammatory response through stress hormones imbalance and circadian rhythm disruption (sleep deprivation or fragmentation), respectively, leading to endothelial dysfunction and platelet aggregation. All these elements can promote ischaemic heart disease

PM2.5 and diesel exhaust exposures have both been implicated in acutely raising blood pressure^50,51^ (*Figure 3*). In meticulously conducted randomized studies, it has been demonstrated that exposure to ultrafine particles, PM2.5, and PM10 increase blood pressure within hours.^39,52^ In contrast, decreased PM2.5 exposure through air filtration tools has shown reduced blood pressure, indicating a cause–effect relationship.^53^ The factors causing this phenomenon may include abrupt changes in autonomic tone, redox stress, changes in vascular stiffness, and endothelial dysfunction.^54,55^

Air pollutants may impair insulin sensitivity and promote the development of overt diabetes mellitus potentially through a variety of pathways including systemic inflammatory insults and oxidative stress.^56–58^ Estimates form the GBD suggest that as much as 22% of the global population attributable fraction of type 2 diabetes may result from air pollution.^59^ In this context, by adding to chronic air pollutant exposure (PM2.5, CO, NO2, and ultrafine particles) the burden of road traffic noise and lack of green spaces, the cumulative risk of multiple exposures appears to be much higher than the risk estimates of any single exposure.^60^

The mechanisms mediating cardiovascular disease in response to air pollution may be viewed as cascading responses beginning with pollutant inhalation in the lung that results in initiating responses, recognition and transmission of these responses, and finally end-organ effector mechanisms.^44,61^ Transmission pathways include biologic intermediates (oxidized lipids, cytokines, microparticles, vasoconstrictors), activated immune cells, autonomic imbalance/afferent neurologic circuits leading to sympathetic and/or hypothalamic-pituitary-adrenal axis (HPAA) activation, and direct translocation of pollutants to the systemic circulation.^52–55^ Nanoparticles in the ultrafine range have been shown to directly leach into the circulation and penetrate atherosclerotic plaque in humans and mice.^62^ Finally, end-organ effector mechanisms responsible for cardiovascular and metabolic responses include: endothelial barrier disruption and/or dysfunction; tissue/organ inflammation; heightened coagulation-thrombosis; and vasoconstriction/increased blood pressure and secondary tissue damage/responses (plaque instability).^56^ Additional mechanisms can include direct disruption of the blood–brain barrier by ultrafine particulate and gaseous co-pollutants which may influence autonomic nervous system as well as resulting in central nervous system inflammation.^63–66^ Oxidative stress and the interplay between interleukin-6 and tissue factor appear to be additional mechanisms in pollution-mediated thrombosis, together with an emerging role for circulating microvesicles and epigenetic changes.^67,68^ Air pollution enhances the thrombotic response, as shown in an experimental model through intratracheal exposure to diesel exhaust particles, which induced platelet activation within an hour^67^; and in human, where inhalation of PM increased platelet–leucocyte aggregates.^69–71^ The thrombotic response appears to be mediated by platelet activation through direct contact in the lung or translocation of ultrafine PM, as well as through mediators released into the circulation as a result of PM-induced lung inflammation^69,70^ (*Figure 3*).

Recent evidence has shown that higher PM2.5 is associated with increased leucopoietic activity, as well as arterial inflammation, even after adjusting for traditional cardiovascular risk factors.^72^ This response is mediated by an enhanced efflux of monocytes from the bone marrow migrating to adipose tissue and arterial wall.^72,73^ Toll-like receptor (TLR) 4 and nicotinamide adenine dinucleotide phosphate (NADPH) oxidase appear to mediate the vascular effects of PM2.5, as well as C-C chemokine receptor type 2 (CCR2), which is critically involved in the mobilization of these cells and adipose tissue inflammation.^54,55,74^ Interestingly, in 631 randomly selected men without overt cardiovascular disease, the exposure to air pollutants was associated with C-reactive protein increase^75^; these results were also observed in ACS patients, demonstrating that C-reactive protein levels present a linear and significant correlation with PM2.5, PM10, and CO exposure.^43^ Pro-oxidant effects further contribute to coronary plaque development and air pollution may finally trigger coronary plaque destabilization. In this context, highly vulnerable coronary plaque features, investigated through intracoronary imaging, were associated with at least 2-year exposure to PM2.5^43^; in stable patients, high levels of PM2.5 were associated with an increased risk of either fibrofatty or necrotic core in newly developed plaques and with a higher risk of total plaque volume progression in the pre-existing plaques.^42^ Lastly, high PM2.5 exposure enhances the occurrence of coronary spasm and portends acute clinical presentation in patients with ischaemia and non-obstructive coronary arteries.^76^

These results should be put into context with data on tobacco smoking and occupational pollution, which have both shown a deep intersection with air pollution, unsafe water, sanitation, and handwashing, as well as with extreme temperatures exposure in determining the risk of death and DALYs.^77^

### Climate change and non-optimal temperatures

Climate change is a major environmental risk factor and is strictly related to air pollution, recognizing in ambient air pollution one of its leading causes.^78^ Of importance, the frequency of heat waves is progressively growing and prolonged exposure to hot temperatures has recently been associated with increased risk of cardiovascular death.^79^ Moreover, heat waves demonstrated a synergistic effect with air pollution determining an increase in MI mortality rates.^80^ Indeed, during prolonged heat waves, thermoregulation is triggered, causing sympathetic activation, increased systemic inflammation, oxidative stress, and endothelial dysfunction, along with metabolic increase and oxygen consumption, thus potentially leading to myocardial ischaemia.^79^

### Soil pollution and water contamination

Soil is responsible for water storing, crop preservation, carbon capturing, and global climate change slowing.^81^ Soil may be polluted by heavy metals, organic chemicals such as pesticides, biological pathogens, and plastic particles, that inevitably contaminate food and drinkable water causing deleterious systemic effects though oxidative stress recognized as a common initiating event.^77,78,81,82^ In particular, water contamination may cause heavy metal accumulation, among which cadmium, lead, and inorganic arsenic have demonstrated strong association with cardiovascular risk factor enhancement, cardiovascular mortality, and adverse cardiac events, particularly IHD and adverse cardiac remodeling.^83–86^

### Light pollution

A novel environmental risk factor is light pollution, defined and measured by the threshold of 14 µcd/m^2^ artificial night-time sky illumination.^87^ This phenomenon comes in many forms, including sky glow, light trespass, glare, and over-illumination. Ninety-nine per cent of the western population lives under light-polluted skies.^88^ In a recent longitudinal study evaluating 58 692 Chinese elders (∼77 years old) followed for a median of 11 years, outdoor light at night at the residential address was associated with a higher risk of IHD hospitalization and mortality, even after adjustment for a wide range of individual and area-level risk factors.^15^ Concordantly, in a cross-sectional study involving elderly individuals in Japan with a mean age of 71.4 years, higher levels of night-time light intensity, measured inside the bedroom, were positively associated with carotid atherosclerosis progression.^16^ Moreover, light at night measured in a home setting was significantly associated with increased night-time blood pressure, as well as hyperglycaemia and obesity, highlighting the role of this pollutant in inducing classical cardiovascular risk factors.^89–91^ Exposure to night-time light may disrupt circadian rhythms which affects a multitude of homeostatic mechanisms that may lead to increased susceptibility through sleep fragmentation, deprivation and stress, and risk factors such as hypertension and obesity (*Figure 3*).^92,93^ Many mediators of circadian rhythms are epigenetic modifiers that influence metabolism through multiple transcriptional pathways.^94–96^ Misalignment between circadian components and environmental factors is thus thought to be an important contributor to non-communicable diseases.^92,97,98^ At a molecular level, autonomous circadian rhythms are generated by a transcription–translation auto-regulatory feedback loop. Epigenetic modifications of the molecular circadian rhythms lead to cardiovascular dysfunction, triggering systemic inflammatory responses, detrimentally affecting the immune system, and increasing superoxide and endothelial NO synthase uncoupling in blood vessels.^55,67,74^ Recent data advance the concept that environmental factors may exert cardiovascular effects via epigenetic alteration of circadian targets.^94,95,99,100^ In an important study comparing chronic ambient inhalational air pollution exposure, PM2.5 caused peripheral insulin resistance, circadian rhythm dysfunction, and metabolic and brown adipose tissue (BAT) dysfunction, similar to light at night (however with no additive interaction between PM2.5 and night-time light).^101^ These phenotypic variations were related with reprogramming of pathways implicated in inflammation, lipid oxidation, and gluconeogenesis, all without changes in body weight. Circadian disruption was evinced by considerable modifications in the rhythmic synthesis of daily corticosteroids, along with changes in amplitude and desynchronization of key circadian and epigenetic regulators such as *Bmal1*, *Clock*, *Per1*, *Per2*, *Cry1*, and *Cry2* in the liver and BAT.^101^ Although there were phenotypic similarities between light at night and PM2.5 exposures, there were also distinct transcriptional and epigenomic differences. By using ATAC-sequencing to detect differentially accessible promoters and enhancers of circadian genes in response to PM2.5, transcriptomic analysis of the liver and BAT revealed extensive but distinctive changes in circadian genes. With increased promoter occupancy by the histone acetyltransferase p300, PM2.5 exposure resulted in a down-regulation of the histone deacetylases 2, 3, and 4. These findings suggest a previously unrecognized role of PM2.5 akin to light exposure in promoting circadian disruption and metabolic dysfunction through epigenetic regulation of circadian targets.

### Acoustic pollution

Transportation noise exposure (road, aircraft, and railway noise), an inevitable consequence of urbanization and globalization, represents a growing threat to human health, precipitating stress reactions and contributing to cardiovascular diseases.^102,103^ As is the case with other emerging environmental risk factors, noise feeds the development of traditional cardiovascular risk factors, above all hypertension (*Figure 3*), often poorly controlled.^19,104,105^

A meta-analysis, including studies on road and aircraft noise, highlighted a 6% higher risk for the occurrence of IHD for every 10 decibels (dB) increase in traffic noise, starting from a threshold of 50 dB, nowadays still considered as low.^106,107^ Furthermore, several studies have shown that the risk of major adverse cardiac events (MACEs) is dose-dependent above 50 dB. Indeed, Saucy *et al.*^18,108^ demonstrated that exposure to night-time aircraft noise >50 dB was significantly associated with mortality for all causes of cardiovascular diseases, mainly IHD.

Animal and human studies provide important insights into the mechanisms by which noise exposure fosters cardiovascular diseases.^20,104^ Noise exposure, especially at night, acts at amygdala level, promotes autonomic imbalance and release of stress hormones with consequent oxidative stress, inflammation, metabolic abnormalities, altered gene expression, and endothelial dysfunction, both in healthy individuals and in subjects with pre-existing cardiovascular diseases.^20,104^ Nuclear imaging demonstrates that noise exposure associates with higher amygdala activation, which mediates arterial inflammation and increased risk for MACEs, even in low-risk patients.^20^ Through circadian disruption, stressors including light, noise, and air pollution may entrain cardiovascular pathways of increased risk such as inflammation and oxidative stress. Noise exposure at night for instance has been associated with increased vascular and cerebral oxidative stress through NOX activation and vascular dysfunction leading to an increased risk of cardiovascular events.^86,109^

## Social stress

A worrisome trend in the 21st century, particularly evident during the COVID-19 pandemic, was a marked risk in mental stress leading in many cases to frank mental illness, including depression, anxiety, and schizophrenia.^110^ Furthermore, there is a growing realization that social stressors, together with other pervasive environmental risk factors, including air pollution and chemical exposures, may result in mental illnesses.

The incidence of mental illness is higher in patients experiencing cardiovascular diseases and the risk of a cardiovascular event is more pronounced in subjects with mental conditions, as demonstrated for those affected by depression, bipolar disorder, or schizophrenia.^111^ Nevertheless, while these conditions only affect a limited portion of the population, mental states associated with significant distress have now become more prevalent, affecting the individual response and perception to stressors.^112^ Nowadays, loneliness and social isolation, especially after the COVID-19 pandemic, have increased and multiple studies have identified them as important risk factors in cardiovascular diseases.^113,114^ Social isolation is perceived as the absence of social relationships and identified as behaviour, while loneliness is a feeling which can be subjective.^115^ Moreover, loneliness is related to a poor quality of social contact that could creep into daily routines and lead to depression and affect mental health.^116^ Of interest, among diabetes patients, loneliness, but not social isolation, is associated with a higher risk of cardiovascular disease and shows an additive interaction with the degree of risk factor control.^117^

**Figure 4:**
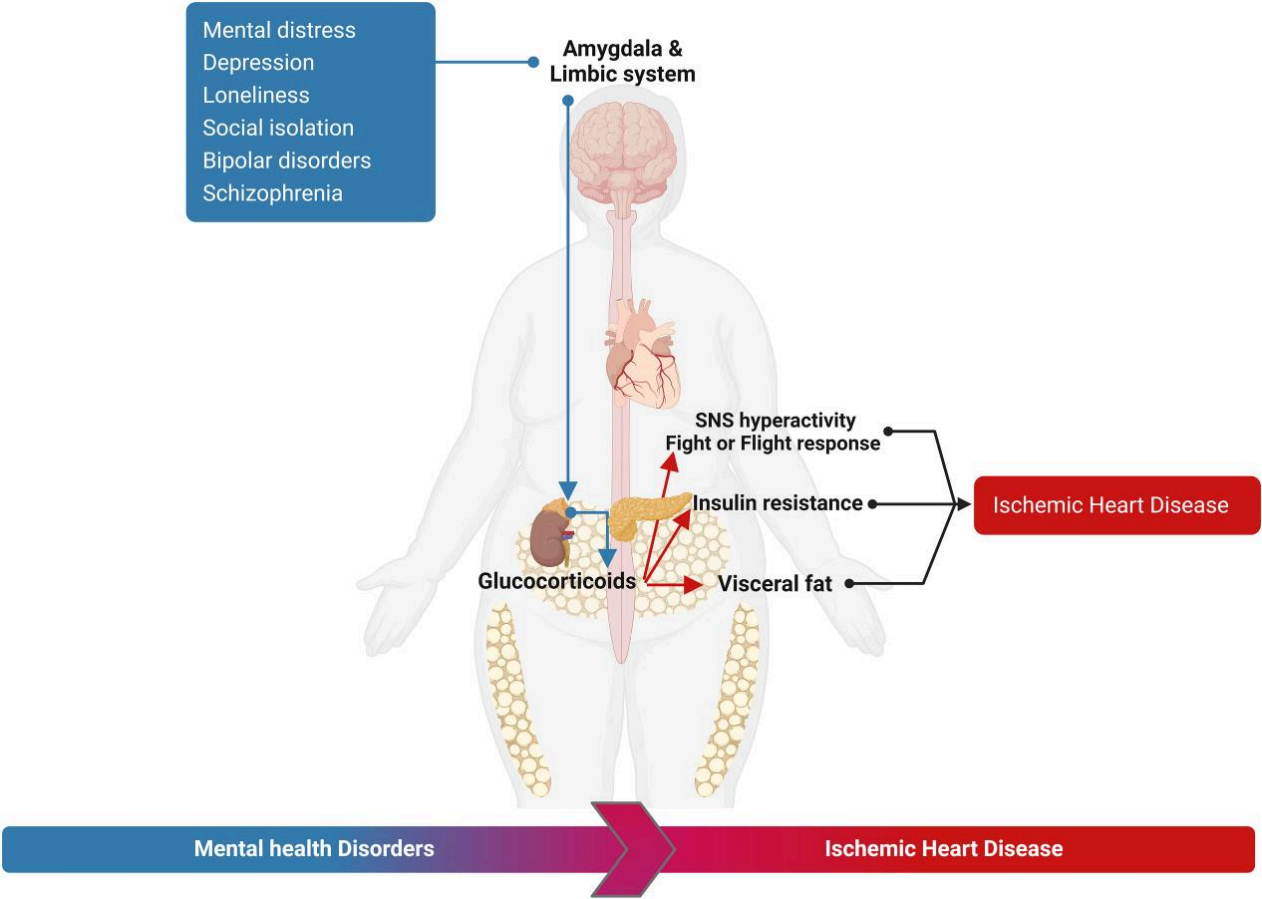
Mental distress, depression, loneliness, and social isolation act as a trigger for ischaemic heart disease through the hypothalamic-pituitary-adrenal axis which induces the production of glucocorticoids by the adrenal cortex, generating insulin resistance and visceral obesity. An imbalance in the sympathetic nervous system promotes the fight or flight response with higher blood pressure and heart rate variability. SNS, sympathetic nervous system

Chronic stress has been repeatedly shown to result in adverse health consequences, irrespective of study characteristics, stressor, outcomes, and confounding factors.^118^ The international INTERHEART case–control study proved that chronic psychosocial factors were significantly related to a doubling risk of acute MI, independently of traditional modifiable risk factors, geographic regions, age, and sex.^119^ However, several residual confounding issues continue to complicate the interpretation of this relationship, given the association of unhealthy lifestyles, including sedentary habits, smoking, and poor diet, that are often highly prevalent.^120^ A major limitation of studies investigating mental stress is the lack of standardized measures for stress quantification.^121^ Cortisol, an index of adrenal activation, involved as a component of the stress response, is a crude plasma indicator of an integrated stress response.^122–124^ More recently, plasma biomarkers have emerged, such as brain-derived neurotrophic factor (BDNF), that possibly reflect propensity to plaque instability.^123,125,126^ Undoubtedly, newer approaches that focus on integrative pathways and shed light on the brain-peripheral responses are needed. Animal models of chronic stress have provided evidence that the neural circuitry regardless of the trigger may share common pathways and elucidate a remarkable preserved cascade of events that is evolutionarily conserved across species.^127^ What is currently abundantly apparent is that the limbic system is a pivotal central component of stress sensing, with the amygdala playing a critical role in anchoring and entraining a diversity of neural centres through the brain efferent pathways. The downstream neural centre of amygdalar response is the hypothalamus that increase sympathetic nervous system (SNS) and initiate activation of the HPAA, with inhibition of the vagal system^123,124^ (*Figure 4*). Hyper-activation of the SNS increases peripheral vascular resistance with higher blood pressure, and lowering heart rate variability.^127–129^ The short-term activation of the HPAA and SNS, while transiently exerting a moderating influence on acute stress pathways such as suppressing a hyperactivated immune response and reducing inflammation, may result in maladaptive pathways chronically, such as immune system dysregulation and chronic low-grade inflammation.^123,124,127–129^ Indeed, a paradigm where chronic stress not only up-regulates stress-associated neurobiological activity but also leads to increased risk factors (obesity, hypertension, and insulin resistance) and heightened arterial inflammation via a neural-immune axis has now been established.^130^ This in turn has been shown to drive higher cardiovascular disease risk independently of traditional risk factors. Indeed, both noise and air pollution result in arterial inflammation with increased amygdalar activation.^74,130^ An integrated mechanism by which a diversity of external triggers including social stress may result in a stereotypical response characterized by amygdalar and HPAA activation and hyper-elicited peripheral sympathetic responses, provides a framework to understand how chronic stress can result in increased risk for cardiovascular disease. A recent study evaluated neighbourhood-level socioeconomic status indices among individuals who had undergone ^18^F-fluorodeoxyglucose positron emission tomography/computed tomography imaging. Lower neighbourhood socioeconomic status (lower income or higher crime) is associated with increased amygdalar activation, arterial inflammation, and subsequent cardiovascular events.^131^

## Infectious diseases

**Figure 5:**
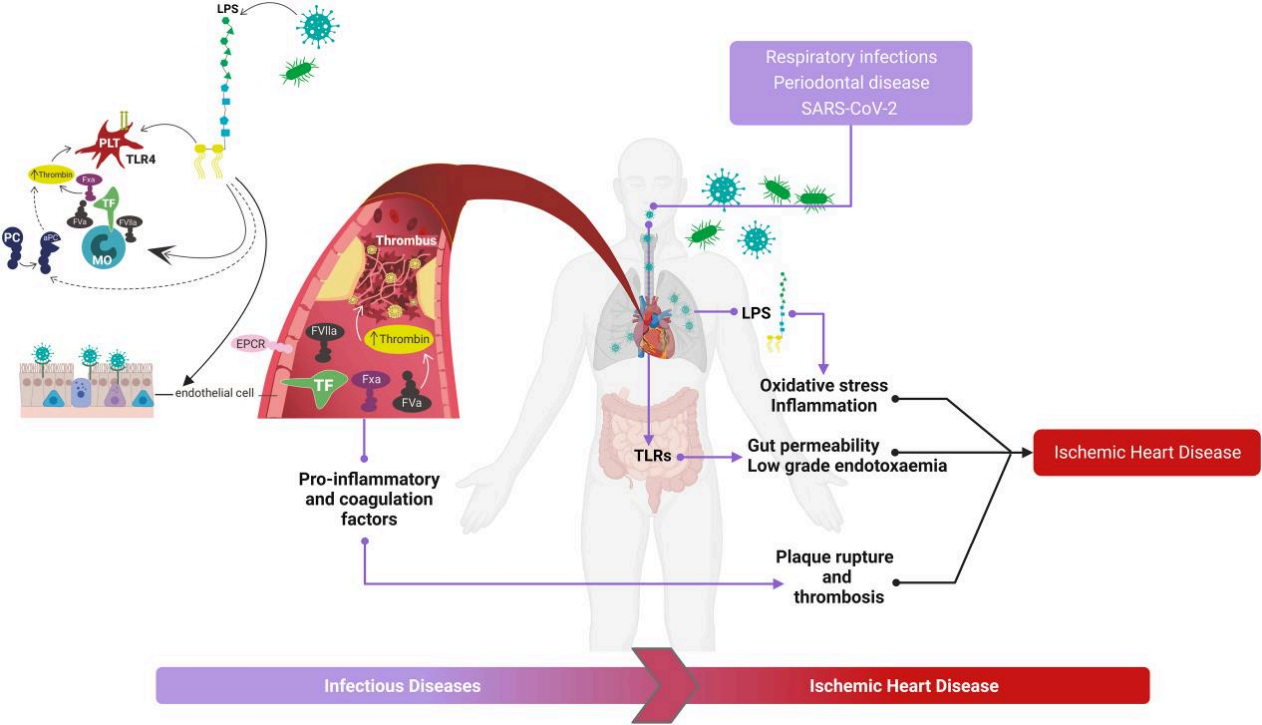
Infectious diseases as risk factors in ischaemic heart disease. Respiratory infections, periodontal diseases, and recently SARS-CoV-2 infection through multiple mechanisms, such as platelet activation, lipopolysaccharide-mediated systemic, inflammation and oxidative stress, mediate ischaemic heart disease progression. Moreover, heightened gut permeability and successive low-grade endotoxaemia, through Toll-like receptors, affect atherosclerotic and thrombotic processes. EPCR, Endothelial protein C receptor; Fxa, Factor Xa; FVa, Factor Va; FVIIa, Factor VIIa; LPS, lipopolysaccharide; MO, monocyte; aPC, activated protein C; PC, protein C; PLT, platelet; TF, tissue factor; TLR, Toll-like receptor; TLR4, Toll-like receptor 4

Infections may represent a risk factor for atherothrombotic cardiovascular events, and this relationship has been investigated in different clinical settings.^132,133^ Respiratory infections, periodontal diseases, *Helicobacter pylori* contamination, *Chlamydia pneumoniae*, and recently the COVID-19 pandemic have all been shown to be related to an increased cardiovascular risk.^132–136^ Nevertheless, the impact of acute respiratory infections on the cardiovascular system has been widely studied in the last few decades,^133,137–139^ characterizing underpinning mechanisms in terms of both acute and chronic consequences.^138^ Direct cardiac myocyte damage, platelet activation, oxidative stress induction, and lipopolysaccharide (LPS)-mediated systemic inflammation have been suggested as the most common pathological events.^140–142^ Concomitantly, enhanced gut permeability and subsequent low-grade endotoxaemia may have effects both on atherosclerotic and thrombotic processes, by binding TLRs, implicated in platelet activation and consequently in thrombus formation^142^ (*Figure 5*). However, platelet activation and aggregation may be directly mediated by pathogen components.^141^ In this context, patients affected by community-acquired pneumonia and presenting acute MI were found to have higher values of mean platelet volume, plasma soluble P-selectin, CD40 ligand, and serum thromboxane B2, as well as increased gut permeability and low-grade endotoxaemia.^143^ In particular, *C. pneumoniae* has been studied in the context of ACS showing that an active, possibly chronic infection might trigger coronary instability.^136^ Moreover, several gram-positive bacteria enhance the thrombotic risk by the formation of platelet–neutrophil complexes.^144^

Lipopolysaccharide plays a pivotal role in plaque formation, progression, and destabilization, through its pro-oxidant properties, which are mediated by the activation of NADPH oxidase-2 (Nox2).^145–147^ Intratracheal administration of LPS in mice induced progression from stable to unstable phenotypes in aortic arch plaques.^148^ Plaque instability was caused by acute inflammation of the arterial wall via leucocyte infiltration and formation of neutrophil extracellular traps (NETs).^148,149^

Influenza and COVID-19 virus present direct and indirect effects on triggering and/or exacerbating IHD presentation, by a vascular endothelial viral infection and the induction of a systemic inflammatory cytokine storm.^134,150^ The direct influenza virus infection of vascular endothelial cells, inducing the epithelial release of a variety of cytokines, chemokines, and adhesion/apoptosis molecules, may accelerate atherosclerosis plaque progression and platelet activation.^151,152^ Systemically, influenza induced-proinflammatory and coagulation factors may be responsible for the increased risk of plaque rupture and thrombosis.^153–155^ Influenza may also enhance thrombus formation by increasing tissue factor and von Willebrand factor (vWF) expression in vascular endothelium,^156^ and plasminogen activator inhibitor-1 levels in plasma,^157^ but also by decreasing protein C activity and by promoting NET formation, finally leading to a higher risk of coronary artery disease.^158^

COVID-19 infection also may directly induce or precipitate cardiovascular events.^134^ Indeed, through the angiotensin-converting enzyme 2 receptor, severe acute respiratory syndrome coronavirus 2 (SARS-CoV-2) may enter vascular smooth cells, endothelial cells, and myocytes, producing a cytopathic effect.^134,159^ The direct effect of SARS-CoV-2 induces endothelial injury, recognized by an increased local release of proinflammatory cytokines and adhesion molecules.^159,160^ Similarly to the influenza virus, the systemic inflammatory cytokine storm in infected patients may trigger cardiac manifestations such as myocarditis, arrhythmias, thromboembolism, heart failure, MI, or multisystem inflammatory syndrome in children.^161^ Indirect effects in severely ill COVID-19 patients are mainly due to vascular hyperpermeability, up-regulation of trypsin, and activation of procoagulant pathways.^162^ In particular, in intensive care unit (ICU)-hospitalized COVID-19 subjects, the levels of vWF antigen were higher compared to non-ICU COVID-19 and to influenza patients.^163,164^ Of importance, many epidemiological studies reported a strong association between SARS-CoV-2 infection, increased PM2.5 exposure, and morbidity and mortality from IHD.^44^ Indeed, several mechanisms crucial for the pathogenesis of SARS-CoV-2 can cross-react and have synergistic effects with those induced by PM2.5, thus exponentially increasing the risk of IHD.

Finally, changes in both gut microbial composition and circulating levels of microbial metabolites have been associated with several human diseases, including cardiovascular diseases. In particular, key gut microbiota-generated metabolites derived from aromatic amino acids have been shown to be independently associated with the occurrence of acute cardiovascular events.^165,166^ Of importance, lifetime exposure to air pollution and other environmental contaminants (i.e. pesticides) can alter the composition and diversity of the gut microbiota and these alterations have been linked to adverse health outcomes,^167,168^ further demonstrating the systemic interactions of these environmental risk factors in determining human diseases.

## The concept of the exposome

**Figure 6:**
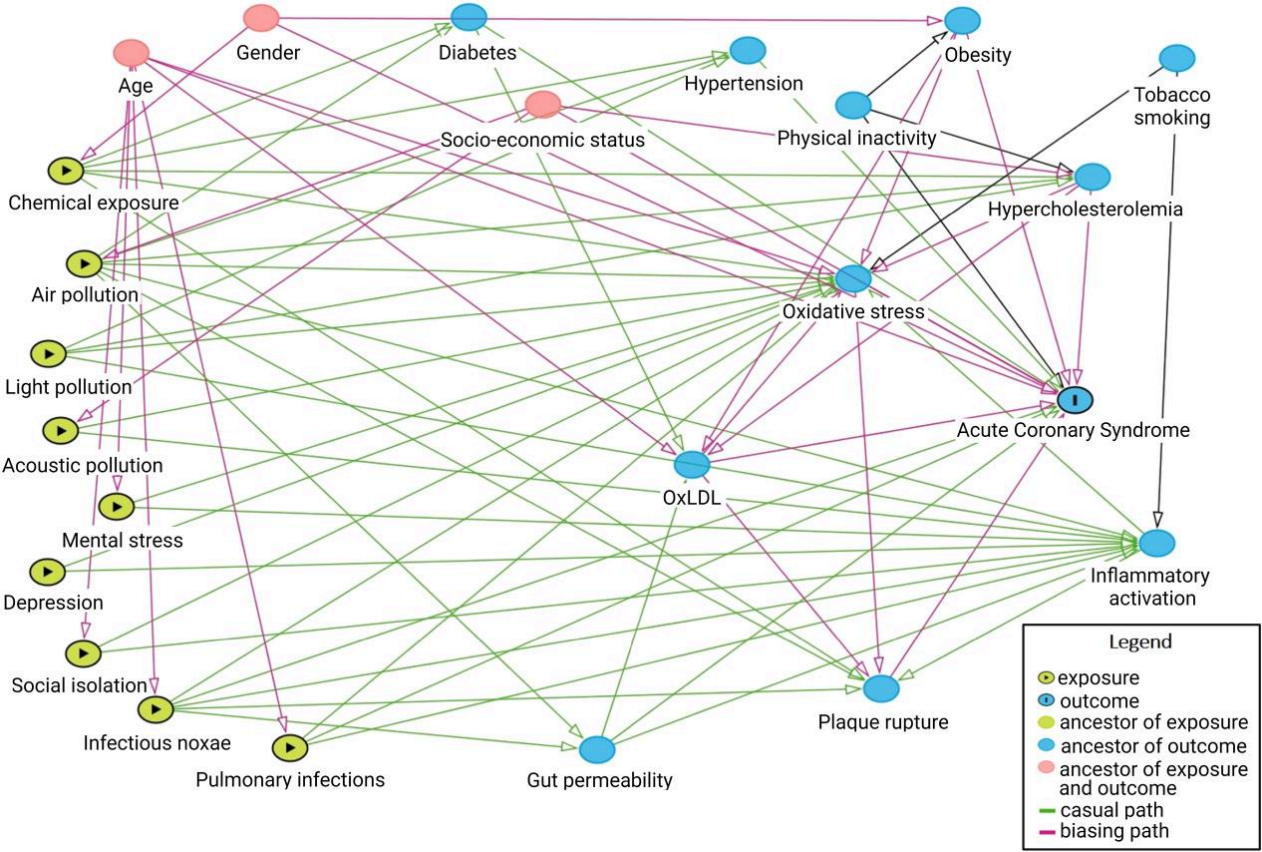
Directed acyclic graph (DAG) illustrating the causal effect of the exposome on ischaemic heart disease (IHD). Being the exposome composed by air pollution, chemical exposure, light pollution, acoustic pollution, mental stress, depression, social isolation, infectious noxae, and pulmonary infections. Highlighting age, gender, and socioeconomic status as confounding factors. The green nodes indicate the exposure of interest, the green lines the exposures effect pathways, the blue node with ‘I’ indicates the outcome of interest, and the blue node without ‘I’ is an intermediate to the effect pathway. The pink nodes show the confounding factors since they are both ancestor of exposure and outcome and therefore, the pink lines identify the biasing path. This DAG was generated through dagitty.net

Based on this increasing awareness of the impact of the natural, built, and social environments on common pathways that heighten susceptibility to chronic non-communicable diseases, the exposome concept has been introduced to identify an emerging field investigating the effects of pan-environmental exposures on human health. In particular, the exposome outlines the harmful biochemical and metabolic changes that occur in the human body due to the combination of different environmental exposures throughout life. In this context, the assessment of exposure-related changes in metabolic and biochemical pathways should be investigated and linked to health outcomes (*Figure 6*), and an approach aiming at the implementation of potential biomarkers, able to integrate the genotypic substrate and including ‘omics’ technologies, may play a crucial role in stratifying the risk of developing cardiovascular events.^78^ Indeed, the exposome concept may not be completely separated by individual genetic predisposition. Since genetic predisposition may explain only a part of the risk in complex diseases such as cancer and IHD, a large proportion of the disease burden may be attributable to environmental stressors and the interplay between the genes and the environment.^78^

The exposome is important to both recognize exposures and define that exposures are not isolated but rather a network generating multiple ways to determine aggregate responses to health. Recent studies corroborate an exaggerated impact of combined exposures, as seen for air and noise pollution, together with lack of green spaces, on metabolic diseases and cardiovascular events.^169,170^ Furthermore, environmental exposures often colocalize with a lower socioeconomic status, social isolation and negative behaviours (unhealthy diet and smoking) in a sort of vicious circle that further increase the risk of cardiovascular events.^171^ The Doughnut model of planetary health provides a unique system view on health.^172^ In this model, social disruption and exceeding planetary boundaries in critical planetary systems, such as climate change, may result in a multitude of exposures including pollutants and social stressors that may result in adverse cardiovascular risk. The built environment, based on a fossil fuel economy of a massive road infrastructure, limited walking opportunities, lack of green spaces and natural vegetation, and a food culture based on animal foods, also culminates in an integrated risk that cannot be obviated by treating conventional risk factors alone.

So far, only a few available studies represented a ‘real’ comprehensive exposome assessment that includes analyses of environmental, lifestyle, and socioeconomic exposures as well as biomarkers of exposures and outcomes.^78^ Thus, our understanding of the cardiovascular health effects and mechanisms of environmental exposures is still limited, because of the lack of large, randomized studies. The European Human Exposome Network (EHEN) is the world’s largest network studying the effect of environmental exposures on health, consisting of nine large-scale research projects, four of which dedicated to cardiovascular outcomes, that will shed light on this topic.^173^

## Potential mitigation strategies

Results from recent pharmacological strategies against lipid, inflammatory, and thrombotic components of the residual cardiovascular risk, which partly reduced but not abated this risk,^174–178^ have further raised the necessity of addressing environmental determinants of the atherosclerotic process. In particular, vulnerable subjects such as patients with previous history of IHD or diabetes mellitus seem to be at higher risk of environmental-driven damage, thus requiring special attention from caring physicians.^78,87^

### Environmental pollution

Clean air, water, and soil together with mitigation of other exposures such as light, and noise pollution, and social well-being are included in the United Nations Sustainable Development Goals, with the aim to achieve a global sustainable development for a better world by 2030.^31,77,78,87,179^ At the core of the exposures is the ongoing anthropogenic activity and reliance on the fossil fuels that powers the multi-layered economy. Thus, satisfactory solutions will require a shift in the current state of a linear ‘fossil fuel’ enabled economy to a ‘reduce-reuse-recycle and regenerate’ circular economy.^180^ Only recently, guidelines from cardiology societies have included air pollution as a risk modifier for cardiovascular disease, highlighting the importance of raising awareness about this major public health threat.^181^ One of the most urgent actions to facilitate this goal is a rapid transition from fossil fuels to clean energy produced from wind and solar power.^31,77,78,87,179^ In the meantime, several policy measures directed at a population level such as reducing car transport use, alternate modes of transport, and incentives for these activities through taxation can be very effective^182^ (*Table 2*).

**Table 2:** Potential mitigation strategies

Environmental exposures	Potential mitigation strategies
Air pollution	Transition from fossil fuels to renewable energy sources (such as wind, tidal, geothermal, and solar) Transportation reforms promoting the use of low- and zero-emission vehicles as well as the restriction of traffic in city centres The use of diesel particle traps, catalytic converters, or alternative fuels (e.g. natural gas and electric cars) Urban landscape reforms: reduction of minimum distances between sources and people, the relocation of traffic sources (e.g. major trafficked roads), and the avoidance of mixed-use areas (industrial-residential) Personal equipment such as face masks and air purifiers Building-level filters such as high efficiency particulate arrestance (HEPA) Behavioural modifications to reduce passive exposures: closing car and home windows, use of cabin air filter for air-conditioning, changing travel routes, staying indoors Lifestyle changes including physical exercise in green areas away from major roadways Planetary diets derived through sustainable sources, climate and health goals
Acoustic pollution	Better traffic management and regulation Implementation of noise reduction protocols Technologies to reduce transportation noise Encourage the use of electric vehicles to reduce traffic noise Design buildings with soundproofing materials and techniques Create more green spaces to act as natural sound buffers
Light pollution	Policies to promote energy conservation and light pollution regulations, such as the ‘dark skies’ legislation Switch off lights when not necessary and use fewer lights when inside Use of automated street lights with motion sensors Keep blinds and drapes closed at night Use night shift settings on all devices Prefer downward facing lights both inside and outside
Social stress	Tracking devices, mental health apps and wearable devices to assess mental activity Need to extend stress-preventive strategies The development of psychosocial interventions aimed at improving mental health and resilience Psychological treatments Mindfulness-based interventions
Infectious diseases	Vaccines against airway infections Personal equipment such as face masks Wash and dry hands regularly and well Sanitation, surface cleaning Cover coughs and sneezes Air cleaning and filtration Mediterranean diet, prebiotics, and probiotics

Awareness of the effects of pan-environmental exposures on cardiovascular health needs to be implemented and, in this regard, personal behaviours are of paramount importance. The easiest mitigation approach to decrease light pollution is to switch off lights when not necessary. Another easy approach is the use of personal air filtration devices. Accordingly, the use of N-95 filters has been shown to reduce blood pressure and markers of inflammation and has been recommended as a personal protective measure to mitigate air pollution exposure for vulnerable individuals by the American Heart Association.^53,183,184^ There is an interest in pharmacotherapies to mitigate air pollution health effects, especially to mitigate the impact of oxidant stress. In this regard, the use of anti-oxidant medications such as vitamin A or E may be somewhat simplistic, given the complexities of redox stress. Planetary diets derived through sustainable sources, procured ethically and composed of natural, non-processed ingredients may help drive simultaneous climate and health goals.^185^ The Mediterranean diet as well as indigenous diets—without artificial processing—can have significant benefits and can also help mitigate the adverse effects of pollution exposure.^186^

### Mental health disorders and social stress

Stress symptoms and psychosocial stressors are considered cardiovascular risk modifiers.^187–189^ Despite the increased awareness on mental diseases and their impact on cardiovascular integrity, data on preventive measures (both pharmacological and psychological) are not univocal, mainly due to the heterogeneity of the population, type of stressors and differences in outcomes evaluated. Furthermore, there is a lack of standardized measures to quantify stress. Although validated questionnaires are still the most commonly used, new technology is providing opportunities to improve stress measurement. Similarly to air pollution tracking devices, mental health apps and wearable devices to assess mental activity may have clinical utility in recording cardiac parameters and relate them to physiological changes (*Table 2*).

### Infectious diseases

Potential therapeutic approaches, both pharmacological and non-pharmacological, have been proposed to reduce the impact of infectious diseases on cardiovascular outcomes^133,142^ (*Table 2*). Non-pharmacological therapies that influence gut permeability, to reduce LPS translocation into circulation, are mainly Mediterranean diet, prebiotics, and probiotics.^190^ Mediterranean diet plays a key role in controlling endotoxaemia, through the reduction of serum zonulin levels and improvement of patients’ metabolic profile.^191,192^ Another strategy to be considered is high dose of fish oil assumption, in order to increase the ratio of n-3 polyunsaturated fatty acids (PUFAs) to n-6 PUFA.^193^ Also statins, largely used in patients with cardiovascular risk factors, may inhibit the proinflammatory effect caused by low-grade endotoxaemia, by increasing NO production and reducing expression of inflammatory molecules.^194^

Vaccines against airway infections represent valuable modulators of cardiovascular risk. The Flu Vaccination ACS study demonstrated a lower cardiovascular mortality in patients with previous MI randomized to receive the influenza vaccine and this effect was significantly evident at 1-year follow-up.^195^ The Influenza Vaccination in Secondary Prevention from Coronary Ischemic Events in Coronary Artery Disease (FLUCAD) trial gave similar positive results.^196^ Furthermore, a meta-analysis from eight randomized clinical trials—influenza vaccination vs. placebo—confirmed a 25% reduction in MACE in patients with recent MI.^197^ Contrasting results are nevertheless available on anti-pneumococcal vaccination.^198^

## Conclusions

A robust body of evidence has proved that environmental, non-traditional risk factors can adversely affect the burden of cardiovascular diseases, being responsible for increased morbidity and reduction of life expectancy. These emerging determinants include exposure to ambient pollution, mental stress, and psychosocial disorders, as well as infectious diseases (*Graphical Abstract*). All these risk factors are not isolated but rather in a network generating multiple ways to determine aggregate responses on human health and in turn amplifying their impact on the cardiovascular system. Although the social awareness of the problem is increasing and the main cardiovascular guidelines are now taking into account the importance of targeting these cardiovascular disease modifiers, there is still a long way to go for implementing preventive and management strategies. In this context, healthcare providers, and public health organizations in general, should be aware of the necessity to deal with this paradigm shift. Finally, further research and interventional trials are needed to deeply investigate how these emerging factors, alone and in combination, impact on cardiovascular system integrity across socioeconomic status, age, sex, ethnicity, and pre-existing conditions.

## Supplementary data

Supplementary data are not available at *European Heart Journal* online.

## Declarations

### Disclosure of Interest

D.L.B. discloses the following relationships—Advisory Board: Angiowave, Bayer, Boehringer Ingelheim, Cardax, CellProthera, Cereno Scientific, Elsevier Practice Update Cardiology, High Enroll, Janssen, Level Ex, McKinsey, Medscape Cardiology, Merck, MyoKardia, NirvaMed, Novo Nordisk, PhaseBio, PLx Pharma, Regado Biosciences, Stasys; Board of Directors: Angiowave (stock options), Boston VA Research Institute, Bristol Myers Squibb (stock), DRS.LINQ (stock options), High Enroll (stock), Society of Cardiovascular Patient Care, TobeSoft; Chair: Inaugural Chair, American Heart Association Quality Oversight Committee; Consultant: Broadview Ventures, Hims; Data Monitoring Committees: Acesion Pharma, Assistance Publique-Hôpitaux de Paris, Baim Institute for Clinical Research (formerly Harvard Clinical Research Institute, for the PORTICO trial, funded by St. Jude Medical, now Abbott), Boston Scientific (Chair, PEITHO trial), Cleveland Clinic (including for the ExCEED trial, funded by Edwards), Contego Medical (Chair, PERFORMANCE 2), Duke Clinical Research Institute, Mayo Clinic, Mount Sinai School of Medicine (for the ENVISAGE trial, funded by Daiichi Sankyo; for the ABILITY-DM trial, funded by Concept Medical), Novartis, Population Health Research Institute; Rutgers University (for the NIH-funded MINT Trial); Honoraria: American College of Cardiology (Senior Associate Editor, Clinical Trials and News, ACC.org; Chair, ACC Accreditation Oversight Committee), Arnold and Porter law firm (work related to Sanofi/Bristol-Myers Squibb clopidogrel litigation), Baim Institute for Clinical Research (formerly Harvard Clinical Research Institute; RE-DUAL PCI clinical trial steering committee funded by Boehringer Ingelheim; AEGIS-II executive committee funded by CSL Behring), Belvoir Publications (Editor in Chief, Harvard Heart Letter), Canadian Medical and Surgical Knowledge Translation Research Group (clinical trial steering committees), CSL Behring (AHA lecture), Cowen and Company, Duke Clinical Research Institute (clinical trial steering committees, including for the PRONOUNCE trial, funded by Ferring Pharmaceuticals), HMP Global (Editor in Chief, *Journal of Invasive Cardiology*), *Journal of the American College of Cardiology* (Guest Editor; Associate Editor), K2P (Co-Chair, interdisciplinary curriculum), Level Ex, Medtelligence/ReachMD (CME steering committees), MJH Life Sciences, Oakstone CME (Course Director, Comprehensive Review of Interventional Cardiology), Piper Sandler, Population Health Research Institute (for the COMPASS operations committee, publications committee, steering committee, and USA national co-leader, funded by Bayer), Slack Publications (Chief Medical Editor, *Cardiology Today’s Intervention*), Society of Cardiovascular Patient Care (Secretary/Treasurer), WebMD (CME steering committees), Wiley (steering committee); Other: *Clinical Cardiology* (Deputy Editor), NCDR-ACTION Registry Steering Committee (Chair), VA CART Research and Publications Committee (Chair); Patent: Sotagliflozin (named on a patent for sotagliflozin assigned to Brigham and Women’s Hospital who assigned to Lexicon; neither I nor Brigham and Women’s Hospital receive any income from this patent); Research Funding: Abbott, Acesion Pharma, Afimmune, Aker Biomarine, Alnylam, Amarin, Amgen, AstraZeneca, Bayer, Beren, Boehringer Ingelheim, Boston Scientific, Bristol-Myers Squibb, Cardax, CellProthera, Cereno Scientific, Chiesi, CinCor, Cleerly, CSL Behring, Eisai, Ethicon, Faraday Pharmaceuticals, Ferring Pharmaceuticals, Forest Laboratories, Fractyl, Garmin, HLS Therapeutics, Idorsia, Ironwood, Ischemix, Janssen, Javelin, Lexicon, Lilly, Medtronic, Merck, Moderna, MyoKardia, NirvaMed, Novartis, Novo Nordisk, Otsuka, Owkin, Pfizer, PhaseBio, PLx Pharma, Recardio, Regeneron, Reid Hoffman Foundation, Roche, Sanofi, Stasys, Synaptic, The Medicines Company, Youngene, 89Bio; Royalties: Elsevier (Editor, Braunwald’s Heart Disease); Site Co-Investigator: Abbott, Biotronik, Boston Scientific, CSI, Endotronix, St. Jude Medical (now Abbott), Philips, SpectraWAVE, Svelte, Vascular Solutions; Trustee: American College of Cardiology; Unfunded Research: FlowCo, Takeda. All other authors have no relevant conflict of interests.

### Data Availability

No data were generated or analysed for this manuscript.

## References

[1] Roth GA , Mensah GA, Johnson CO, Addolorato G, Ammirati E, Baddour LM, et al Global burden of cardiovascular diseases and risk factors, 1990–2019: update from the GBD 2019 study. J Am Coll Cardiol 2020;76:2982–3021. 10.1016/j.jacc.2020.11.010 33309175 PMC7755038

[2] Knuuti J , Wijns W, Saraste A, Capodanno D, Barbato E, Funck-Brentano C, et al 2019 ESC guidelines for the diagnosis and management of chronic coronary syndromes. Eur Heart J 2020;41:407–77. 10.1093/eurheartj/ehz425 31504439

[3] Gutiérrez E , Flammer AJ, Lerman LO, Elízaga J, Lerman A, Fernández-Avilés F. Endothelial dysfunction over the course of coronary artery disease. Eur Heart J 2013;34:3175–81. 10.1093/eurheartj/eht351 24014385 PMC3814514

[4] Andersson C , Johnson AD, Benjamin EJ, Levy D, Vasan RS. 70-Year legacy of the Framingham Heart Study. Nat Rev Cardiol 2019;16:687–98. 10.1038/s41569-019-0202-5 31065045

[5] Andersson C , Nayor M, Tsao CW, Levy D, Vasan RS. Framingham Heart Study: JACC Focus Seminar, 1/8. J Am Coll Cardiol 2021;77:2680–92. 10.1016/j.jacc.2021.01.059 34045026

[6] Gibbons GH , Seidman CE, Topol EJ. Conquering atherosclerotic cardiovascular disease—50 years of progress. N Engl J Med 2021;384:785–8. 10.1056/NEJMp2033115 33657686

[7] Figtree GA , Vernon ST, Hadziosmanovic N, Sundström J, Alfredsson J, Arnott C, et al Mortality in STEMI patients without standard modifiable risk factors: a sex-disaggregated analysis of SWEDEHEART registry data. Lancet 2021;397:1085–94. 10.1016/S0140-6736(21)00272-5 33711294

[8] Yusuf S , Joseph P, Rangarajan S, Islam S, Mente A, Hystad P, et al Modifiable risk factors, cardiovascular disease, and mortality in 155 722 individuals from 21 high-income, middle-income, and low-income countries (PURE): a prospective cohort study. Lancet 2020;395:795–808. 10.1016/S0140-6736(19)32008-2. Epub 2019 Sep 3. Erratum in: Lancet. 2020 Mar 7; 395(10226):784. PMC8006904 31492503

[9] Downward GS , van Nunen EJHM, Kerckhoffs J, Vineis P, Brunekreef B, Boer JMA, et al Long-term exposure to ultrafine particles and incidence of cardiovascular and cerebrovascular disease in a prospective study of a Dutch cohort. Environ Health Perspect 2018;126:127007. 10.1289/EHP3047 30566375 PMC6371648

[10] Turner MC , Jerrett M, Pope CA 3rd, Krewski D, Gapstur SM, Diver WR, et al Long-term ozone exposure and mortality in a large prospective study. Am J Respir Crit Care Med 2016;193:1134–42. 10.1164/rccm.201508-1633OC 26680605 PMC4872664

[11] Kaufman JD , Adar SD, Barr RG, Budoff M, Burke GL, Curl CL, et al Association between air pollution and coronary artery calcification within six metropolitan areas in the USA (the Multi-Ethnic Study of Atherosclerosis and Air Pollution): a longitudinal cohort study. Lancet 2016;388:696–704. 10.1016/S0140-6736(16)00378-0. Erratum in: Lancet. 2016 Aug 13; 388(10045):660. 27233746 PMC5019949

[12] Cesaroni G , Forastiere F, Stafoggia M, Andersen ZJ, Badaloni C, Beelen R, et al Long term exposure to ambient air pollution and incidence of acute coronary events: prospective cohort study and meta-analysis in 11 European cohorts from the ESCAPE Project. BMJ 2014;348:f7412. 10.1136/bmj.f7412 24452269 PMC3898420

[13] Pope CA 3rd , Burnett RT, Thun MJ, Calle EE, Krewski D, Ito K, et al Lung cancer, cardiopulmonary mortality, and long-term exposure to fine particulate air pollution. JAMA 2002;287:1132–41. 10.1001/jama.287.9.1132 11879110 PMC4037163

[14] Xu YX , Yu Y, Huang Y, Wan YH, Su PY, Tao FB, et al Exposure to bedroom light pollution and cardiometabolic risk: a cohort study from Chinese young adults. Environ Pollut 2022;294:118628. 10.1016/j.envpol.2021.118628 34883146

[15] Sun S , Cao W, Ge Y, Ran J, Sun F, Zeng Q, et al Outdoor light at night and risk of coronary heart disease among older adults: a prospective cohort study. Eur Heart J 2021;42:822–30. 10.1093/eurheartj/ehaa846 33205210

[16] Obayashi K , Yamagami Y, Tatsumi S, Kurumatani N, Saeki K. Indoor light pollution and progression of carotid atherosclerosis: a longitudinal study of the HEIJO-KYO cohort. Environ Int 2019;133:105184. 10.1016/j.envint.2019.105184 31648154

[17] Obayashi K , Saeki K, Iwamoto J, Ikada Y, Kurumatani N. Association between light exposure at night and nighttime blood pressure in the elderly independent of nocturnal urinary melatonin excretion. Chronobiol Int 2014;31:779–86. 10.3109/07420528.2014.900501 24673296

[18] Saucy A , Schäffer B, Tangermann L, Vienneau D, Wunderli JM, Röösli M. Does night-time aircraft noise trigger mortality? A case-crossover study on 24886 cardiovascular deaths. Eur Heart J 2021;42:835–43. 10.1093/eurheartj/ehaa957 PMC7897463 33245107

[19] Kupcikova Z , Fecht D, Ramakrishnan R, Clark C, Cai YS. Road traffic noise and cardiovascular disease risk factors in UK Biobank. Eur Heart J 2021;42:2072–84. 10.1093/eurheartj/ehab121 33733673 PMC8169156

[20] Osborne MT , Radfar A, Hassan MZO, Abohashem S, Oberfeld B, Patrich T, et al A neurobiological mechanism linking transportation noise to cardiovascular disease in humans. Eur Heart J 2020;41:772–82. 10.1093/eurheartj/ehz820 31769799 PMC7006229

[21] Correia AW , Peters JL, Levy JI, Melly S, Dominici F. Residential exposure to aircraft noise and hospital admissions for cardiovascular diseases: multi-airport retrospective study. BMJ 2013;347:f5561. 10.1136/bmj.f5561 24103538 PMC3805481

[22] Gan Y , Gong Y, Tong X, Sun H, Cong Y, Dong X, et al Depression and the risk of coronary heart disease: a meta-analysis of prospective cohort studies. BMC Psychiatry 2014;14:371. 10.1186/s12888-014-0371-z 25540022 PMC4336481

[23] Nabi H , Kivimäki M, Batty GD, Shipley MJ, Britton A, Brunner EJ, et al Increased risk of coronary heart disease among individuals reporting adverse impact of stress on their health: the Whitehall II prospective cohort study. Eur Heart J 2013;34:2697–705. 10.1093/eurheartj/eht216 23804585 PMC3766148

[24] Richardson S , Shaffer JA, Falzon L, Krupka D, Davidson KW, Edmondson D. Meta-analysis of perceived stress and its association with incident coronary heart disease. Am J Cardiol 2012;110:1711–6. 10.1016/j.amjcard.2012.08.004 22975465 PMC3511594

[25] Russ TC , Stamatakis E, Hamer M, Starr JM, Kivimäki M, Batty GD. Association between psychological distress and mortality: individual participant pooled analysis of 10 prospective cohort studies. BMJ 2012;345:e4933. 10.1136/bmj.e4933 22849956 PMC3409083

[26] Janszky I , Ahnve S, Lundberg I, Hemmingsson T. Early-onset depression, anxiety, and risk of subsequent coronary heart disease: 37-year follow-up of 49,321 young Swedish men. J Am Coll Cardiol 2010;56:31–7. 10.1016/j.jacc.2010.03.033 20620714

[27] Pieralli F , Vannucchi V, Nozzoli C, Augello G, Dentali F, De Marzi G, et al Correction to: acute cardiovascular events in patients with community acquired pneumonia: results from the observational prospective FADOI-ICECAP study. BMC Infect Dis 2021;21:195. 10.1186/s12879-021-05891-5. Erratum for: BMC Infect Dis. 2021 Jan 25; 21(1):116. 33607965 PMC7893753

[28] Violi F , Cangemi R, Falcone M, Taliani G, Pieralli F, Vannucchi V, et al Cardiovascular complications and short-term mortality risk in community-acquired pneumonia. Clin Infect Dis 2017;64:1486–93. 10.1093/cid/cix164. Erratum in: Clin Infect Dis. 2017 Oct 15; 65(8):1431–1433. 28205683

[29] Cangemi R , Calvieri C, Falcone M, Bucci T, Bertazzoni G, Scarpellini MG, et al Relation of cardiac complications in the early phase of community-acquired pneumonia to long-term mortality and cardiovascular events. Am J Cardiol 2015;116:647–51. 10.1016/j.amjcard.2015.05.028 26089009

[30] Corrales-Medina VF , Musher DM, Wells GA, Chirinos JA, Chen L, Fine MJ. Cardiac complications in patients with community-acquired pneumonia: incidence, timing, risk factors, and association with short-term mortality. Circulation 2012;125:773–81. 10.1161/CIRCULATIONAHA.111.040766 22219349

[31] Cosselman KE , Navas-Acien A, Kaufman JD. Environmental factors in cardiovascular disease. Nat Rev Cardiol 2015;12:627–42. 10.1038/nrcardio.2015.152 26461967

[32] Bhatnagar A . Environmental determinants of cardiovascular disease. Circ Res 2017;121:162–80. 10.1161/CIRCRESAHA.117.306458 28684622 PMC5777598

[33] Global Buden of Disease (GBD) . https://www.healthdata.org/research-analysis/gbd (4 September 2023, date last accessed).

[34] Lelieveld J , Pozzer A, Pöschl U, Fnais M, Haines A, Münzel T. Loss of life expectancy from air pollution compared to other risk factors: a worldwide perspective. Cardiovasc Res 2020;116:1910–7. 10.1093/cvr/cvaa025 32123898 PMC7449554

[35] GBD 2019 . https://vizhub.healthdata.org/gbd-compare/#0 (4 September 2023, date last accessed).

[36] Wild CP . Complementing the genome with an “exposome”: the outstanding challenge of environmental exposure measurement in molecular epidemiology. Cancer Epidemiol Biomarkers Prev 2005;14:1847–50. 10.1158/1055-9965 16103423

[37] Matter MA , Paneni F, Libby P, Frantz S, Stähli BE, Templin C, et al Inflammation in acute myocardial infarction: the good, the bad and the ugly. Eur Heart J 2024;45:89–103. 10.1093/eurheartj/ehad486 37587550 PMC10771378

[38] Rajagopalan S , Landrigan PJ. Pollution and the heart. N Engl J Med 2021;385:1881–92. 10.1056/NEJMra2030281 34758254

[39] Al-Kindi SG , Brook RD, Biswal S, Rajagopalan S. Environmental determinants of cardiovascular disease: lessons learned from air pollution. Nat Rev Cardiol 2020;17:656–72. 10.1038/s41569-020-0371-2 32382149 PMC7492399

[40] Landrigan PJ , Fuller R, Acosta NJR, Adeyi O, Arnold R, Basu NN, et al The Lancet Commission on pollution and health. Lancet 2018;391:462–512. 10.1016/S0140-6736(17)32345-0 29056410

[41] Lancet T . UK air pollution and public health. Lancet 2017;389:1860. 10.1016/S0140-6736(17)31271-0 28513437

[42] Yang S , Lee SP, Park JB, Lee H, Kang SH, Lee SE, et al PM2.5 concentration in the ambient air is a risk factor for the development of high-risk coronary plaques. Eur Heart J Cardiovasc Imaging 2019;20:1355–64. 10.1093/ehjci/jez209 31410457

[43] Montone RA , Camilli M, Russo M, Termite C, La Vecchia G, Iannaccone G, et al Air pollution and coronary plaque vulnerability and instability: an optical coherence tomography study. JACC Cardiovasc Imaging 2022;15:325–42. 10.1016/j.jcmg.2021.09.008 34656488

[44] Montone RA , Rinaldi R, Bonanni A, Severino A, Pedicino D, Crea F, et al Impact of air pollution on ischemic heart disease: evidence, mechanisms, clinical perspectives. Atherosclerosis 2023;366:22–31. 10.1016/j.atherosclerosis.2023.01.013 36696748

[45] Russo M , Rinaldi R, Camilli M, Bonanni A, Caffè A, Basile M, et al Air pollution and plaque healing in acute coronary syndromes. Eur Heart J 2023;44:2403–5. 10.1093/eurheartj/ehad319 37264700

[46] Jacobs L , Emmerechts J, Hoylaerts MF, Mathieu C, Hoet PH, Nemery B, et al Traffic air pollution and oxidized LDL. PLoS One 2011;6:e16200. 10.1371/journal.pone.0016200 21283820 PMC3023773

[47] Ossoli A , Cetti F, Gomaraschi M. Air pollution: another threat to HDL function. Int J Mol Sci 2023;24:317. 10.3390/ijms24010317 PMC9820244 36613760

[48] Li J , Zhou C, Xu H, Brook RD, Liu S, Yi T, et al Ambient air pollution is associated with HDL (high-density lipoprotein) dysfunction in healthy adults. Arterioscler Thromb Vasc Biol 2019;39:513–22. 10.1161/ATVBAHA.118.311749 30700134

[49] Yao H , Lv J. Statin attenuated myocardial inflammation induced by PM2.5 in rats. Acta Cardiol Sin 2017;33:637–45. 10.6515/ACS20170518A 29167617 PMC5694928

[50] Cai Y , Zhang B, Ke W, Feng B, Lin H, Xiao J, et al Associations of short-term and long-term exposure to ambient air pollutants with hypertension: a systematic review and meta-analysis. Hypertension 2016;68:62–70. 10.1161/HYPERTENSIONAHA.116.07218 27245182

[51] Szyszkowicz M , Rowe BH, Brook RD. Even low levels of ambient air pollutants are associated with increased emergency department visits for hypertension. Can J Cardiol 2012;28:360–6. 10.1016/j.cjca.2011.06.011 21944840

[52] Rajagopalan S , Al-Kindi SG, Brook RD. Air pollution and cardiovascular disease: JACC State-of-the-Art Review. J Am Coll Cardiol 2018;72:2054–70. 10.1016/j.jacc.2018.07.099 30336830

[53] Rajagopalan S , Brauer M, Bhatnagar A, Bhatt DL, Brook JR, Huang W, et al Personal-level protective actions against particulate matter air pollution exposure: a scientific statement from the American Heart Association. Circulation 2020;142:e411–31. 10.1161/CIR.0000000000000931 33150789

[54] Munzel T , Sorensen M, Gori T, Schmidt FP, Rao X, Brook FR, et al Environmental stressors and cardio-metabolic disease: part II—mechanistic insights. Eur Heart J 2017;38:557–64. 10.1093/eurheartj/ehw294 27460891 PMC5381593

[55] Munzel T , Gori T, Al-Kindi S, Deanfield J, Lelieveld J, Daiber A, et al Effects of gaseous and solid constituents of air pollution on endothelial function. Eur Heart J 2018;39:3543–50. 10.1093/eurheartj/ehy481 30124840 PMC6174028

[56] Hill BG , Rood B, Ribble A, Haberzettl P. Fine particulate matter (PM2.5) inhalation-induced alterations in the plasma lipidome as promoters of vascular inflammation and insulin resistance. Am J Physiol Heart Circ Physiol 2021;320:H1836–50. 10.1152/ajpheart.00881.2020 33666505 PMC8163652

[57] Bowe B , Xie Y, Li T, Yan Y, Xian H, Al-Aly Z. The 2016 global and national burden of diabetes mellitus attributable to PM2.5 air pollution. Lancet Planet Health 2018;2:e301–12. 10.1016/S2542-5196(18)30140-2 30074893

[58] Rajagopalan S , Brook RD. Air pollution and type 2 diabetes: mechanistic insights. Diabetes 2012;61:3037–45. 10.2337/db12-0190 23172950 PMC3501850

[59] GBD 2019 Diabetes and Air Pollution Collaborators . Estimates, trends, and drivers of the global burden of type 2 diabetes attributable to PM2.5 air pollution, 1990–2019: an analysis of data from the Global Burden of Disease Study 2019. Lancet Planet Health 2022;6:e586–600. 10.1016/S2542-5196(22)00122-X 35809588 PMC9278144

[60] Sørensen M , Poulsen AH, Hvidtfeldt UA, Brandt J, Frohn LM, Ketzel M, et al Air pollution, road traffic noise and lack of greenness and risk of type 2 diabetes: a multi-exposure prospective study covering Denmark. Environ Int 2022;170:107570. 10.1016/j.envint.2022.107570 36334460

[61] Gangwar RS , Bevan GH, Palanivel R, Das L, Rajagopalan S. Oxidative stress pathways of air pollution mediated toxicity: recent insights. Redox Biol 2020;34:101545. 10.1016/j.redox.2020.101545 32505541 PMC7327965

[62] Miller MR , Raftis JB, Langrish JP, McLean SG, Samutrtai P, Connell SP, et al Inhaled nanoparticles accumulate at sites of vascular disease. ACS Nano 2017;11:4542–52. 10.1021/acsnano.6b08551 28443337 PMC5444047

[63] Calderon-Garciduenas L , Solt AC, Henriquez-Roldan C, Torres-Jardon R, Nuse B, Herritt L, et al Long-term air pollution exposure is associated with neuroinflammation, an altered innate immune response, disruption of the blood–brain barrier, ultrafine particulate deposition, and accumulation of amyloid beta-42 and alpha-synuclein in children and young adults. Toxicol Pathol 2008;36:289–310. 10.1177/0192623307313011 18349428

[64] Block ML , Elder A, Auten RL, Bilbo SD, Chen H, Chen JC, et al The outdoor air pollution and brain health workshop. Neurotoxicology 2012;33:972–84. 10.1016/j.neuro.2012.08.014 22981845 PMC3726250

[65] Aragon MJ , Topper L, Tyler CR, Sanchez B, Zychowski K, Young T, et al Serum-borne bioactivity caused by pulmonary multiwalled carbon nanotubes induces neuroinflammation via blood–brain barrier impairment. Proc Natl Acad Sci U S A 2017;114:E1968–76. 10.1073/pnas.1616070114 28223486 PMC5347541

[66] Mumaw CL , Levesque S, McGraw C, Robertson S, Lucas S, Stafflinger JE, et al Microglial priming through the lung–brain axis: the role of air pollution-induced circulating factors. FASEB J 2016;30:1880–91. 10.1096/fj.201500047 26864854 PMC4836369

[67] Hantrakool S , Kumfu S, Chattipakorn SC, Chattipakorn N. Effects of particulate matter on inflammation and thrombosis: past evidence for future prevention. Int J Environ Res Public Health 2022;19:8771. 10.3390/ijerph19148771 35886623 PMC9317970

[68] Rider CF , Carlsten C. Air pollution and DNA methylation: effects of exposure in humans. Clin Epigenetics 2019;11:131. 10.1186/s13148-019-0713-2 31481107 PMC6724236

[69] Franchini M , Mannucci PM. Thrombogenicity and cardiovascular effects of ambient air pollution. Blood 2011;118:2405–12. 10.1182/blood-2011-04-343111 21666054

[70] Franchini M , Guida A, Tufano A, Coppola A. Air pollution, vascular disease and thrombosis: linking clinical data and pathogenic mechanisms. J Thromb Haemost 2012;10:2438–51. 10.1111/jth.12006 23006215

[71] Nemmar A , Hoet PH, Vandervoort P, Dinsdale D, Nemery B, Hoylaerts MF. Enhanced peripheral thrombogenicity after lung inflammation is mediated by platelet–leukocyte activation: role of P-selectin. J Thromb Haemost 2007;5:1217–26. 10.1111/j.1538-7836.2007.02557.x 17403095

[72] Abohashem S , Osborne MT, Dar T, Naddaf N, Abbasi T, Ghoneem A, et al A leucopoietic-arterial axis underlying the link between ambient air pollution and cardiovascular disease in humans. Eur Heart J 2021;42:761–72. 10.1093/eurheartj/ehaa982 33428721 PMC7882372

[73] Goto Y , Ishii H, Hogg JC, Shih CH, Yatera K, Vincent R, et al Particulate matter air pollution stimulates monocyte release from the bone marrow. Am J Respir Crit Care Med 2004;170:891–7. 10.1164/rccm.200402-235OC 15256391

[74] Provoost S , Maes T, Joos GF, Tournoy KG. Monocyte-derived dendritic cell recruitment and allergic T(H)2 responses after exposure to diesel particles are CCR2 dependent. J Allergy Clin Immunol 2012;129:483–91. 10.1016/j.jaci.2011.07.051 21906792

[75] Peters A , Fröhlich M, Döring A, Immervoll T, Wichmann HE, Hutchinson WL, et al Particulate air pollution is associated with an acute phase response in men; results from the MONICA-Augsburg Study. Eur Heart J 2001;22:1198–204. 10.1053/euhj.2000.2483 11440492

[76] Camilli M , Russo M, Rinaldi R, Caffè A, La Vecchia G, Bonanni A, et al Air pollution and coronary vasomotor disorders in patients with myocardial ischemia and unobstructed coronary arteries. J Am Coll Cardiol 2022;80:1818–28. 10.1016/j.jacc.2022.08.744 36049556

[77] Fuller R , LandriganPJ, BalakrishnanK, BathanG, Bose-O’ReillyS, BrauerM, et al Pollution and health: a progress update. Lancet Planet Health2022;6:e535–47. 10.1016/S2542-5196(22)00090-035594895

[78] Münzel T , Sørensen M, Hahad O, Nieuwenhuijsen M, Daiber A. The contribution of the exposome to the burden of cardiovascular disease. Nat Rev Cardiol 2023;20:651–69. 10.1038/s41569-023-00873-3 37165157

[79] Alahmad B , Khraishah H, Royé D, Vicedo-Cabrera AM, Guo Y, Papatheodorou SI, et al Associations between extreme temperatures and cardiovascular cause-specific mortality: results from 27 countries. Circulation 2023;147:35–46. 10.1161/CIRCULATIONAHA.122.061832 36503273 PMC9794133

[80] Xu R , Huang S, Shi C, Wang R, Liu T, Li Y, et al Extreme temperature events, fine particulate matter, and myocardial infarction mortality. Circulation 2023;148:312–23. 10.1161/CIRCULATIONAHA.122.063504 37486993

[81] Li H , Kilgallen AB, Münzel T, Wolf E, Lecour S, Schulz R, et al Influence of mental stress and environmental toxins on circadian clocks: implications for redox regulation of the heart and cardioprotection. Br J Pharmacol 2020;177:5393–412. 10.1111/bph.14949 31833063 PMC7680009

[82] Tellez-Plaza M , Jones MR, Dominguez-Lucas A, Guallar E, Navas-Acien A. Cadmium exposure and clinical cardiovascular disease: a systematic review. Curr Atheroscler Rep 2013;15:356. 10.1007/s11883-013-0356-2 23955722 PMC3858820

[83] Messner B , Knoflach M, Seubert A, Ritsch A, Pfaller K, Henderson B, et al Cadmium is a novel and independent risk factor for early atherosclerosis mechanisms and in vivo relevance. Arterioscler Thromb Vasc Biol 2009;29:1392–8. 10.1161/ATVBAHA.109.190082 19556524

[84] Navas-Acien A , Guallar E, Silbergeld EK, Rothenberg SJ. Lead exposure and cardiovascular disease—a systematic review. Environ Health Perspect 2007;115:472–82. 10.1289/ehp.9785 17431501 PMC1849948

[85] Pichler G , Grau-Perez M, Tellez-Plaza M, Umans J, Best L, Cole S, et al Association of arsenic exposure with cardiac geometry and left ventricular function in young adults. Circ Cardiovasc Imaging 2019;12:e009018. 10.1161/CIRCIMAGING.119.009018 31060373 PMC6668025

[86] Münzel T , Miller MR, Sørensen M, Lelieveld J, Daiber A, Rajagopalan S. Reduction of environmental pollutants for prevention of cardiovascular disease: it’s time to act. Eur Heart J 2020;41:3989–97. 10.1093/eurheartj/ehaa745 33141181 PMC7672530

[87] Münzel T , Hahad O, Sørensen M, Lelieveld J, Duerr GD, Nieuwenhuijsen M, et al Environmental risk factors and cardiovascular diseases: a comprehensive expert review. Cardiovasc Res 2022;118:2880–902. 10.1093/cvr/cvab316 34609502 PMC9648835

[88] Falchi F , Cinzano P, Duriscoe D, Kyba CC, Elvidge CD, Baugh K, et al The new world atlas of artificial night sky brightness. Sci Adv 2016;2:e1600377. 10.1126/sciadv.1600377 27386582 PMC4928945

[89] Obayashi K , Saeki K, Iwamoto J, Ikada Y, Kurumatani N. Independent associations of exposure to evening light and nocturnal urinary melatonin excretion with diabetes in the elderly. Chronobiol Int 2014;31:394–400. 10.3109/07420528.2013.864299 24328728

[90] Zheng R , Xin Z, Li M, Wang T, Xu M, Lu J, et al Outdoor light at night in relation to glucose homoeostasis and diabetes in Chinese adults: a national and cross-sectional study of 98,658 participants from 162 study sites. Diabetologia 2023;66:336–45. 10.1007/s00125-022-05819-x 36372821

[91] McFadden E , Jones ME, Schoemaker MJ, Ashworth A, Swerdlow AJ. The relationship between obesity and exposure to light at night: cross-sectional analyses of over 100,000 women in the Breakthrough Generations Study. Am J Epidemiol 2014;180:245–50. 10.1093/aje/kwu117 24875371

[92] Crnko S , Du Pré BC, Sluijter JPG, Van Laake LW. Circadian rhythms and the molecular clock in cardiovascular biology and disease. Nat Rev Cardiol 2019;16:437–47. 10.1038/s41569-019-0167-4 30796369

[93] Anisimov VN , Vinogradova IA, Panchenko AV, Popovich IG, Zabezhinski MA. Light-at-night-induced circadian disruption, cancer and aging. Curr Aging Sci 2013;5:170–7. 10.2174/1874609811205030002 23237593

[94] Masri S , Zocchi L, Katada S, Mora E, Sassone-Corsi P. The circadian clock transcriptional complex: metabolic feedback intersects with epigenetic control. Ann N Y Acad Sci 2012;1264:103–9. 10.1111/j.1749-6632.2012.06649.x 22834651 PMC3464365

[95] Masri S , Sassone-Corsi P. The circadian clock: a framework linking metabolism, epigenetics and neuronal function. Nat Rev Neurosci 2013;14:69–75. 10.1038/nrn3393 23187814 PMC5720680

[96] Hernandez-Rosas F , Lopez-Rosas CA, Saavedra-Velez MV. Disruption of the molecular circadian clock and cancer: an epigenetic link. Biochem Genet 2020;58:189–209. 10.1007/s10528-019-09938-w 31552565

[97] Liu H , Chen A. Roles of sleep deprivation in cardiovascular dysfunctions. Life Sci 2019;219:231–7. 10.1016/j.lfs.2019.01.006 30630005

[98] Steffens S , Winter C, Schloss MJ, Hidalgo A, Weber C, Soehnlein O. Circadian control of inflammatory processes in atherosclerosis and its complications. Arterioscler Thromb Vasc Biol 2017;37:1022–8. 10.1161/atvbaha.117.309374 28450299

[99] Breton CV , Marsit CJ, Faustman E, Nadeau K, Goodrich JM, Dolinoy DC, et al Small-magnitude effect sizes in epigenetic end points are important in children’s environmental health studies: the children’s environmental health and disease prevention research center’s epigenetics working group. Environ Health Perspect 2017;125:511–26. 10.1289/EHP595 28362264 PMC5382002

[100] Wang T , Pehrsson EC, Purushotham D, Li D, Zhuo X, Zhang B, et al The NIEHS TaRGET II Consortium and environmental epigenomics. Nat Biotechnol 2018;36:225–7. 10.1038/nbt.4099 29509741 PMC5991835

[101] Palanivel R , Vinayachandran V, Biswal S, Deiuliis JA, Padmanabhan R, Park B, et al Exposure to air pollution disrupts circadian rhythm through alterations in chromatin dynamics. iScience 2020;23:101728. 10.1016/j.isci.2020.101728 33241196 PMC7672280

[102] Crea F . Light and noise pollution and socioeconomic status: the risk factors individuals cannot change. Eur Heart J 2021;42:801–4. 10.1093/eurheartj/ehab074 33611398

[103] Kempen EV , Casas M, Pershagen G, Foraster M. WHO environmental noise guidelines for the European Region: a systematic review on environmental noise and cardiovascular and metabolic effects: a summary. Int J Environ Res Public Health 2018;15:379. 10.3390/ijerph15020379 29470452 PMC5858448

[104] Mannucci PM , Ancona C. Noise and air pollution as triggers of hypertension. Eur Heart J 2021;42:2085–7. 10.1093/eurheartj/ehab104 33748836

[105] Hahad O , Rajagopalan S, Lelieveld J, Sørensen M, Frenis K, Daiber A, et al Noise and air pollution as risk factors for hypertension: part I—epidemiology. Hypertension 2023;80:175–1383. 10.1161/HYPERTENSIONAHA.122.18732 PMC10330192 37073726

[106] Vienneau D , Schindler C, Perez L, Probst-Hensch N, Röösli M. The relationship between transportation noise exposure and ischemic heart disease: a meta-analysis. Environ Res 2015;138:372–80. 10.1016/j.envres.2015.02.023 25769126

[107] Münzel T , Sørensen M, Schmidt F, Schmidt E, Steven S, Kröller-Schön S, et al The adverse effects of environmental noise exposure on oxidative stress and cardiovascular risk. Antioxid Redox Signal 2018;28:873–908. 10.1089/ars.2017.7118 29350061 PMC5898791

[108] Saucy A , Schäffer B, Tangermann L, Vienneau D, Wunderli JM, Röösli M. Individual aircraft noise exposure assessment for a case-crossover study in Switzerland. Int J Environ Res Public Health 2020;17:3011. 10.3390/ijerph17093011 32357482 PMC7246478

[109] Kröller-Schön S , Daiber A, Steven S, Oelze M, Frenis K, Kalinovic S, et al Crucial role for Nox2 and sleep deprivation in aircraft noise-induced vascular and cerebral oxidative stress, inflammation, and gene regulation. Eur Heart J 2018;39:3528–39. 10.1093/eurheartj/ehy333 29905797 PMC6174027

[110] COVID-19 Mental Disorders Collaborators . Global prevalence and burden of depressive and anxiety disorders in 204 countries and territories in 2020 due to the COVID-19 pandemic. Lancet 2021;398:1700–12. 10.1016/S0140-6736(21)02143-7 34634250 PMC8500697

[111] Correll CU , Solmi M, Veronese N, Bortolato B, Rosson S, Santonastaso P, et al Prevalence, incidence and mortality from cardiovascular disease in patients with pooled and specific severe mental illness: a large-scale meta-analysis of 3,211,768 patients and 113,383,368 controls. World Psychiatry 2017;16:163–80. 10.1002/wps.20420 28498599 PMC5428179

[112] Levine GN , Cohen BE, Commodore-Mensah Y, Fleury J, Huffman JC, Khalid U, et al Psychological health, well-being, and the mind-heart-body connection: a scientific statement from the American Heart Association. Circulation 2021;143:e763–83. 10.1161/CIR.0000000000000947 33486973

[113] Valtorta NK , Kanaan M, Gilbody S, Ronzi S, Hanratty B. Loneliness and social isolation as risk factors for coronary heart disease and stroke: systematic review and meta-analysis of longitudinal observational studies. Heart 2016;102:1009–16. 10.1136/heartjnl-2015-308790 27091846 PMC4941172

[114] Golaszewski NM , LaCroix AZ, Godino JG, Allison MA, Manson JE, King JJ, et al Evaluation of social isolation, loneliness, and cardiovascular disease among older women in the US. JAMA Netw Open 2022;5:e2146461. 10.1001/jamanetworkopen.2021.46461 35107574 PMC8811637

[115] Christiansen J , Lund R, Qualter P, Andersen CM, Pedersen SS, Lasgaard M. Loneliness, social isolation, and chronic disease outcomes. Ann Behav Med 2021;55:203–15. 10.1093/abm/kaaa044 32865550

[116] Mushtaq R , Shoib S, Shah T, Mushtaq S. Relationship between loneliness, psychiatric disorders and physical health? A review on the psychological aspects of loneliness. J Clin Diagn Res 2014;8:WE01–4. 10.7860/JCDR/2014/10077.4828 25386507 PMC4225959

[117] Wang X , Ma H, Li X, Heianza Y, Fonseca V, Qi L. Joint association of loneliness and traditional risk factor control and incident cardiovascular disease in diabetes patients. Eur Heart J 2023;44:2583–91. 10.1093/eurheartj/ehad306 37385629 PMC10361009

[118] Mariotti A . The effects of chronic stress on health: new insights into the molecular mechanisms of brain-body communication. Future Sci OA 2015;1:FSO23. 10.4155/fso.15.21 28031896 PMC5137920

[119] Rosengren A , Hawken S, Ounpuu S, Sliwa K, Zubaid M, Almahmeed WA, et al Association of psychosocial risk factors with risk of acute myocardial infarction in 11119 cases and 13648 controls from 52 countries (the INTERHEART study): case–control study. Lancet 2004;364:953–62. 10.1016/S0140-6736(04)17019-0 15364186

[120] Chandola T , Brunner E, Marmot M. Chronic stress at work and the metabolic syndrome: prospective study. BMJ 2006;332:521–5. 10.1136/bmj.38693.435301.80 16428252 PMC1388129

[121] Epel ES , Crosswell AD, Mayer SE, Prather AA, Slavich GM, Puterman E, et al More than a feeling: a unified view of stress measurement for population science. Front Neuroendocrinol 2018;49:146–69. 10.1016/j.yfrne.2018.03.001 29551356 PMC6345505

[122] Kivimäki M , Steptoe A. Effects of stress on the development and progression of cardiovascular disease. Nat Rev Cardiol 2018;15:215–29. 10.1038/nrcardio.2017.189 29213140

[123] Russell G , Lightman S. The human stress response. Nat Rev Endocrinol 2019;15:525–34. 10.1038/s41574-019-0228-0 31249398

[124] Elenkov IJ , Chrousos GP. Stress system-organization, physiology and immunoregulation. Neuroimmunomodulation 2006;13:257–67. 10.1159/000104853 17709947

[125] Dhama K , Latheef SK, Dadar M, Samad HA, Munjal A, Khandia R, et al Biomarkers in stress related diseases/disorders: diagnostic, prognostic, and therapeutic values. Front Mol Biosci 2019;6:91. 10.3389/fmolb.2019.00091 31750312 PMC6843074

[126] Montone RA , Camilli M, Del Buono MG, Russo M, Rinaldi R, Canonico F, et al Brain-derived neurotrophic factor in patients with acute coronary syndrome. Transl Res 2021;231:39–54. 10.1016/j.trsl.2020.11.006 33221484

[127] Herman JP , Ostrander MM, Mueller NK, Figueiredo H. Limbic system mechanisms of stress regulation: hypothalamo-pituitary-adrenocortical axis. Prog Neuropsychopharmacol Biol Psychiatry 2005;29:1201–13. 10.1016/j.pnpbp.2005.08.006 16271821

[128] Miller GE , Cohen S, Ritchey AK. Chronic psychological stress and the regulation of pro-inflammatory cytokines: a glucocorticoid-resistance model. Health Psychol 2002;21:531–41. 10.1037//0278-6133.21.6.531 12433005

[129] Fleshner M , Exosomes CC. DAMPs and miRNA: features of stress physiology and immune homeostasis. Trends Immunol 2017;38:768–76. 10.1016/j.it.2017.08.002 28838855 PMC5624844

[130] Osborne MT , Shin LM, Mehta NN, Pitman RK, Fayad ZA, Tawakol A. Disentangling the links between psychosocial stress and cardiovascular disease. Circ Cardiovasc Imaging 2020;13:e010931. 10.1161/CIRCIMAGING.120.010931 32791843 PMC7430065

[131] Tawakol A , Osborne MT, Wang Y, Hammed B, Tung B, Patrich T, et al Stress-associated neurobiological pathway linking socioeconomic disparities to cardiovascular disease. J Am Coll Cardiol 2019;73:3243–55. 10.1016/j.jacc.2019.04.042 31248544 PMC6767929

[132] Szwed P , Gąsecka A, Zawadka M, Eyileten C, Postuła M, Mazurek T, et al Infections as novel risk factors of atherosclerotic cardiovascular diseases: pathophysiological links and therapeutic implications. J Clin Med 2021;10:2539. 10.3390/jcm10122539 34201137 PMC8229654

[133] Pedicino D , Giglio AF, Galiffa VA, Cialdella P, Trotta F, Graziani F, et al Infections, immunity and atherosclerosis: pathogenic mechanisms and unsolved questions. Int J Cardiol 2013;166:572–83. 10.1016/j.ijcard.2012.05.098 22727974

[134] Evans PC , Rainger GE, Mason JC, Guzik TJ, Osto E, Stamataki Z, et al Endothelial dysfunction in COVID-19: a position paper of the ESC Working Group for Atherosclerosis and Vascular Biology, and the ESC Council of Basic Cardiovascular Science. Cardiovasc Res 2020;116:2177–84. 10.1093/cvr/cvaa230 32750108 PMC7454368

[135] Liuzzo G , Volpe M. SARS-CoV-2 infection markedly increases long-term cardiovascular risk. Eur Heart J 2022;43:1899–900. 10.1093/eurheartj/ehac168 35362024 PMC9383629

[136] Liuzzo G , Ciervo A, Niccoli G, Mancini F, Fusco B, Montone RA, et al *Chlamydia pneumoniae* in coronary atherosclerotic plaques and coronary instability. Int J Cardiol 2011;147:176–8. 10.1016/j.ijcard.2010.12.029 21215478

[137] Nguyen JL , Yang W, Ito K, Matte TD, Shaman J, Kinney PL. Seasonal influenza infections and cardiovascular disease mortality. JAMA Cardiol 2016;1:274–81. 10.1001/jamacardio.2016.0433 27438105 PMC5158013

[138] Corrales-Medina VF , Madjid M, Musher DM. Role of acute infection in triggering acute coronary syndromes. Lancet Infect Dis 2010;10:83–92. 10.1016/S1473-3099(09)70331-7 20113977

[139] Warren-Gash C , Bhaskaran K, Hayward A, Leung GM, Lo SV, Wong CM, et al Circulating influenza virus, climatic factors, and acute myocardial infarction: a time series study in England and Wales and Hong Kong. J Infect Dis 2011;203:1710–8. 10.1093/infdis/jir171 21606529 PMC3100509

[140] Brown AO , Millett ER, Quint JK, Orihuela CJ. Cardiotoxicity during invasive pneumococcal disease. Am J Respir Crit Care Med 2015;191:739–45. 10.1164/rccm.201411-1951PP 25629643 PMC4407487

[141] Cangemi R , Casciaro M, Rossi E, Calvieri C, Bucci T, Calabrese CM, et al Platelet activation is associated with myocardial infarction in patients with pneumonia. J Am Coll Cardiol 2014;64:1917–25. 10.1016/j.jacc.2014.07.985 25444147

[142] Violi F , Cammisotto V, Bartimoccia S, Pignatelli P, Carnevale R, Nocella C. Gut-derived low-grade endotoxaemia, atherothrombosis and cardiovascular disease. Nat Rev Cardiol 2023;20:24–37. 10.1038/s41569-022-00737-2 35840742 PMC9284488

[143] Cangemi R , Pignatelli P, Carnevale R, Bartimoccia S, Nocella C, Falcone M, et al Low-grade endotoxemia, gut permeability and platelet activation in community-acquired pneumonia. J Infect 2016;73:107–14. 10.1016/j.jinf.2016.05.013 27288596

[144] Wienkamp AK , Erpenbeck L, Rossaint J. Platelets in the NETworks interweaving inflammation and thrombosis. Front Immunol 2022;13:953129. 10.3389/fimmu.2022.953129 35979369 PMC9376363

[145] Carnevale R , Nocella C, Petrozza V, Cammisotto V, Pacini L, Sorrentino V, et al Localization of lipopolysaccharide from *Escherichia coli* into human atherosclerotic plaque. Sci Rep 2018;8:3598. 10.1038/s41598-018-22076-4 29483584 PMC5826929

[146] den Dekker WK , Cheng C, Pasterkamp G, Duckers HJ. Toll like receptor 4 in atherosclerosis and plaque destabilization. Atherosclerosis 2010;209:314–20. 10.1016/j.atherosclerosis.2009.09.075 19900676

[147] Batty M , Bennett MR, Yu E. The role of oxidative stress in atherosclerosis. Cells 2022;11:3843. 10.3390/cells11233843 36497101 PMC9735601

[148] Jaw JE , Tsuruta M, Oh Y, Schipilow J, Hirano Y, Ngan DA, et al Lung exposure to lipopolysaccharide causes atherosclerotic plaque destabilisation. Eur Respir J 2016;48:205–15. 10.1183/13993003.00972-2015 27009170

[149] Schumski A , Ortega-Gómez A, Wichapong K, Winter C, Lemnitzer P, Viola JR, et al Endotoxinemia accelerates atherosclerosis through electrostatic charge-mediated monocyte adhesion. Circulation 2021;143:254–66. 10.1161/CIRCULATIONAHA.120.046677 33167684 PMC7914394

[150] Liu X , Yang N, Tang J, Liu S, Luo D, Duan Q, et al Downregulation of angiotensin-converting enzyme 2 by the neuraminidase protein of influenza A (H1N1) virus. Virus Res 2014;185:64–71. 10.1016/j.virusres.2014.03.010 24662240 PMC7114376

[151] Haidari M , Wyde PR, Litovsky S, Vela D, Ali M, Casscells SW, et al Influenza virus directly infects, inflames, and resides in the arteries of atherosclerotic and normal mice. Atherosclerosis 2010;208:90–6. 10.1016/j.atherosclerosis.2009.07.028 19665123

[152] Wang S , Le TQ, Kurihara N, Chida J, Cisse Y, Yano M, et al Influenza virus-cytokine-protease cycle in the pathogenesis of vascular hyperpermeability in severe influenza. J Infect Dis 2010;202:991–1001. 10.1086/656044 20731583 PMC7537608

[153] García-Sastre A , Durbin RK, Zheng H, Palese P, Gertner R, Levy DE, et al The role of interferon in influenza virus tissue tropism. J Virol 1998;72:8550–8. 10.1128/JVI.72.11.8550-8558.1998 9765393 PMC110265

[154] Fatkhullina AR , Peshkova IO, Koltsova EK. The role of cytokines in the development of atherosclerosis. Biochemistry 2016;81:1358–70. 10.1134/S0006297916110134 27914461 PMC5471837

[155] Damjanovic D , Small CL, Jeyanathan M, McCormick S, Xing Z. Immunopathology in influenza virus infection: uncoupling the friend from foe. Clin Immunol 2012;144:57–69. 10.1016/j.clim.2012.05.005 22673491

[156] Sugiyama MG , Gamage A, Zyla R, Armstrong SM, Advani S, Advani A, et al Influenza virus infection induces platelet-endothelial adhesion which contributes to lung injury. J Virol 2016;90:1812–23. 10.1128/JVI.02599-15 26637453 PMC4733979

[157] Keller TT , van der Sluijs KF, de Kruif MD, Gerdes VE, Meijers JC, Florquin S, et al Effects on coagulation and fibrinolysis induced by influenza in mice with a reduced capacity to generate activated protein C and a deficiency in plasminogen activator inhibitor type 1. Circ Res 2006;99:1261–9. 10.1161/01.RES.0000250834.29108.1a 17068293

[158] Zhu L , Liu L, Zhang Y, Pu L, Liu J, Li X, et al High level of neutrophil extracellular traps correlates with poor prognosis of severe influenza A infection. J Infect Dis 2018;217:428–37. 10.1093/infdis/jix475 29325098

[159] Varga Z , Flammer AJ, Steiger P, Haberecker M, Andermatt R, Zinkernagel AS, et al Endothelial cell infection and endotheliitis in COVID-19. Lancet 2020;395:1417–8. 10.1016/S0140-6736(20)30937-5 32325026 PMC7172722

[160] Lindner D , Fitzek A, Bräuninger H, Aleshcheva G, Edler C, Meissner K, et al Association of cardiac infection with SARS-CoV-2 in confirmed COVID-19 autopsy cases. JAMA Cardiol 2020;5:1281–5. 10.1001/jamacardio.2020.3551 32730555 PMC7385672

[161] Ahmed M , Advani S, Moreira A, Zoretic S, Martinez J, Chorath K, et al Multisystem inflammatory syndrome in children: a systematic review. EClinicalMedicine 2020;26:100527. 10.1016/j.eclinm.2020.100527 32923992 PMC7473262

[162] Ackermann M , Verleden SE, Kuehnel M, Haverich A, Welte T, Laenger F, et al Pulmonary vascular endothelialitis, thrombosis, and angiogenesis in COVID-19. N Engl J Med 2020;383:120–8. 10.1056/NEJMoa2015432 32437596 PMC7412750

[163] Goshua G , Pine AB, Meizlish ML, Chang CH, Zhang H, Bahel P, et al Endotheliopathy in COVID-19-associated coagulopathy: evidence from a single-centre, cross-sectional study. Lancet Haematol 2020;7:e575–82. 10.1016/S2352-3026(20)30216-7 32619411 PMC7326446

[164] Akiyama R , Komori I, Hiramoto R, Isonishi A, Matsumoto M, Fujimura Y. H1n1 influenza (swine flu)-associated thrombotic microangiopathy with a markedly high plasma ratio of von Willebrand factor to ADAMTS13. Intern Med 2011;50:643–7. 10.2169/internalmedicine.50.4620 21422695

[165] Nemet I , Li XS, Haghikia A, Li L, Wilcox J, Romano KA, et al Atlas of gut microbe-derived products from aromatic amino acids and risk of cardiovascular morbidity and mortality. Eur Heart J 2023;44:3085–96. 10.1093/eurheartj/ehad333 37342006 PMC10481777

[166] Pisano E , Bugli F, Severino A, Pedicino D, Paroni Sterbini F, Martini C, et al Microbial signature of plaque and gut in acute coronary syndrome. Sci Rep 2023;13:14775. 10.1038/s41598-023-41867-y 37679428 PMC10484905

[167] Filardo S , Di Pietro M, Protano C, Antonucci A, Vitali M, Sessa R. Impact of air pollution on the composition and diversity of human gut microbiota in general and vulnerable populations: a systematic review. Toxics 2022;10:579. 10.3390/toxics10100579 36287859 PMC9607944

[168] Thompson KN , Oulhote Y, Weihe P, Wilkinson JE, Ma S, Zhong H, et al Effects of lifetime exposures to environmental contaminants on the adult gut microbiome. Environ Sci Technol 2022;56:16985–95. 10.1021/acs.est.2c03185 36394280

[169] Osborne MT , Abohashem S, Naddaf N, Abbasi T, Zureigat H, Mezue K, et al The combined effect of air and transportation noise pollution on atherosclerotic inflammation and risk of cardiovascular disease events. J Nucl Cardiol 2023;30:665–79. 10.1007/s12350-022-03003-7 35915324 PMC9889575

[170] Poulsen AH , Sørensen M, Hvidtfeldt UA, Christensen JH, Brandt J, Frohn LM, et al Concomitant exposure to air pollution, green space, and noise and risk of stroke: a cohort study from Denmark. Lancet Reg Health Eur 2023;31:100655. 10.1016/j.lanepe.2023.100655 37265507 PMC10230828

[171] GBD 2019 Risk Factors Collaborators . Global burden of 87 risk factors in 204 countries and territories, 1990–2019: a systematic analysis for the Global Burden of Disease Study 2019. Lancet 2020;396:1223–49. 10.1016/S0140-6736(20)30752-2 33069327 PMC7566194

[172] Raworth K . A Doughnut for the Anthropocene: humanity’s compass in the 21st century. Lancet Planet Health 2017;1:e48–9. 10.1016/S2542-5196(17)30028-1 29851576

[173] European Human Exposome Network . https://www.humanexposome.eu/.

[174] Imazio M , Nidorf M. Colchicine and the heart. Eur Heart J 2021;42:2745–60. 10.1093/eurheartj/ehab221 33961006 PMC8294843

[175] Fiolet ATL , Opstal TSJ, Mosterd A, Eikelboom JW, Jolly SS, Keech AC, et al Efficacy and safety of low-dose colchicine in patients with coronary disease: a systematic review and meta-analysis of randomized trials. Eur Heart J 2021;42:2765–75. 10.1093/eurheartj/ehab115. Erratum in: Eur Heart J. 2021 May 23; PMID: 33769515. 33769515

[176] Strandberg TE , Kovanen PT. Coronary artery disease: ‘gout’ in the artery? Eur Heart J 2021;42:2761–4. 10.1093/eurheartj/ehab276 34050656 PMC8845033

[177] Ridker PM , MacFadyen JG, Glynn RJ, Bradwin G, Hasan AA, Rifai N. Comparison of interleukin-6, C-reactive protein, and low-density lipoprotein cholesterol as biomarkers of residual risk in contemporary practice: secondary analyses from the Cardiovascular Inflammation Reduction Trial. Eur Heart J 2020;41:2952–61. 10.1093/eurheartj/ehaa160 32221587 PMC7453833

[178] Koenig W . Persistent inflammatory residual risk despite aggressive cholesterol-lowering therapy: further evidence fuelling the dual target concept. Eur Heart J 2020;41:2962–4. 10.1093/eurheartj/ehaa186 32268369

[179] Lelieveld J , Klingmüller K, Pozzer A, Pöschl U, Fnais M, Daiber A, et al Cardiovascular disease burden from ambient air pollution in Europe reassessed using novel hazard ratio functions. Eur Heart J 2019;40:1590–6. 10.1093/eurheartj/ehz135 30860255 PMC6528157

[180] Corona B , Shen L, Reike D, Carreón JR, Worrell E. Towards sustainable development through the circular economy—a review and critical assessment on current circularity metrics. Resour Conserv Recycl 2019;151:104498. 10.1016/j.resconrec.2019.104498

[181] Visseren FLJ , Mach F, Smulders YM, Carballo D, Koskinas KC, Bäck M, et al 2021 ESC guidelines on cardiovascular disease prevention in clinical practice. Eur Heart J 2021;42:3227–337. 10.1093/eurheartj/ehab484 34458905

[182] Kaufman JD , Elkind MSV, Bhatnagar A, Koehler K, Balmes JR, Sidney S, et al Guidance to reduce the cardiovascular burden of ambient air pollutants: a policy statement from the American Heart Association. Circulation 2020;142:e432–47. 10.1161/CIR.0000000000000930 33147996

[183] World Health Organization . WHO Global Air Quality Guidelines: Particulate Matter (PM2.5 and PM10), Ozone, Nitrogen Dioxide, Sulfur Dioxide and Carbon Monoxide. Geneva: WHO, 2021. https://www.who.int/publications/i/item/9789240034228. 34662007

[184] Newman JD , Bhatt DL, Rajagopalan S, Balmes JR, Brauer M, Breysse PN, et al Cardiopulmonary impact of particulate air pollution in high-risk populations: JACC State-of-the-Art Review. J Am Coll Cardiol 2020;76:2878–94. 10.1016/j.jacc.2020.10.020 33303078 PMC8040922

[185] Willett W , Rockström J, Loken B, Springmann M, Lang T, Vermeulen S, et al Food in the Anthropocene: the EAT-Lancet Commission on healthy diets from sustainable food systems. Lancet 2019;393:447–92. 10.1016/S0140-6736(18)31788-4 30660336

[186] Lim CC , Hayes RB, Ahn J, Shao Y, Silverman DT, Jones RR, et al Mediterranean diet and the association between air pollution and cardiovascular disease mortality risk. Circulation 2019;139:1766–75. 10.1161/CIRCULATIONAHA.118.035742 30700142 PMC6453737

[187] Satyjeet F , Naz S, Kumar V, Aung NH, Bansari K, Irfan S, et al Psychological stress as a risk factor for cardiovascular disease: a case–control study. Cureus 2020;12:e10757. 10.7759/cureus.10757 33150108 PMC7603890

[188] Park JW , Howe CJ, Dionne LA, Scarpaci MM, Needham BL, Sims M, et al Social support, psychosocial risks, and cardiovascular health: using harmonized data from the Jackson Heart Study, Mediators of Atherosclerosis in South Asians Living in America Study, and Multi-Ethnic Study of Atherosclerosis. SSM Popul Health 2022;20:101284. 10.1016/j.ssmph.2022.101284 36387018 PMC9646650

[189] Hussain M , Howell JL, Peek MK, Stowe RP, Zawadzki MJ. Psychosocial stressors predict lower cardiovascular disease risk among Mexican-American adults living in a high-risk community: findings from the Texas City Stress and Health Study. PLoS One 2021;16:e0257940. 10.1371/journal.pone.0257940 34618834 PMC8496861

[190] De Filippis F , Pellegrini N, Vannini L, Jeffery IB, La Storia A, Laghi L, et al High-level adherence to a Mediterranean diet beneficially impacts the gut microbiota and associated metabolome. Gut 2016;65:1812–21. 10.1136/gutjnl-2015-309957 26416813

[191] Bailey MA , Holscher HD. Microbiome-mediated effects of the Mediterranean diet on inflammation. Adv Nutr 2018;9:193–206. 10.1093/advances/nmy013 29767701 PMC5952955

[192] Bartimoccia S , Cammisotto V, Nocella C, Del Ben M, D’Amico A, Castellani V, et al Extra virgin olive oil reduces gut permeability and metabolic endotoxemia in diabetic patients. Nutrients 2022;14:2153. 10.3390/nu14102153 35631294 PMC9145083

[193] Dourado E , Ferro M, Sousa Guerreiro C, Fonseca JE. Diet as a modulator of intestinal microbiota in rheumatoid arthritis. Nutrients 2020;12:3504. 10.3390/nu12113504 33202579 PMC7696404

[194] Zhang S , Zhang Y, Ahsan MZ, Yuan Y, Liu G, Han X, et al Atorvastatin attenuates cold-induced hypertension by preventing gut barrier injury. J Cardiovasc Pharmacol 2019;74:143–51. 10.1097/FJC.0000000000000690 31310598

[195] Gurfinkel EP , de la Fuente R L, Mendiz O, Mautner B. Flu vaccination in acute coronary syndromes and planned percutaneous coronary interventions (FLUVACS) study. Eur Heart J 2004;25:25–31. 10.1016/j.ehj.2003.10.018 14683739

[196] Ciszewski A , Bilinska ZT, Brydak LB, Kepka C, Kruk M, Romanowska M, et al Influenza vaccination in secondary prevention from coronary ischaemic events in coronary artery disease: FLUCAD study. Eur Heart J 2008;29:1350–8. 10.1093/eurheartj/ehm581 18187561

[197] Maniar YM , Al-Abdouh A, Michos ED. Influenza vaccination for cardiovascular prevention: further insights from the IAMI trial and an updated meta-analysis. Curr Cardiol Rep 2022;24:1327–35. 10.1007/s11886-022-01748-8 35876953 PMC9310360

[198] Jaiswal V , Ang SP, Lnu K, Ishak A, Pokhrel NB, Chia JE, et al Effect of pneumococcal vaccine on mortality and cardiovascular outcomes: a systematic review and meta-analysis. J Clin Med 2022;11:3799. 10.3390/jcm11133799 35807082 PMC9267914

